# Population PK/PD Modeling and Simulation to Predict the Comparative Long-Term Efficacy of Olokizumab in Rheumatoid Arthritis Patients

**DOI:** 10.3390/jcm15197660

**Published:** 2026-10-02

**Authors:** Yuri Kosinsky, Ivan Azarov, Kirill Zhudenkov, Kirill Peskov, Sergey Grishin, Alina Egorova, Yuliya Berezovskaya, Maria Lemak, Mikhail Samsonov, Eugen Feist

**Affiliations:** 1 M&S Decisions FZ-LLC, Dubai 1318, United Arab Emirates; yuri.kosinsky@msdecisions.tech (Y.K.); ivan.azarov@msdecisions.tech (I.A.); kirill.zhudenkov@msdecisions.tech (K.Z.); kirill.peskov@msdecisions.tech (K.P.); 2 Research Center of Model-Informed Drug Development, I.M. Sechenov First Moscow State Medical University, Moscow 119991, Russia; 3 Marchuk Institute of Numerical Mathematics of the Russian Academy of Sciences, Moscow 119333, Russia; 4 Medical Department, R-Pharm JSC, Moscow 119421, Russia; sa.grishin@rpharm.ru (S.G.); an.egorova@rpharm.ru (A.E.); yv.berezovskaya@rpharm.ru (Y.B.); samsonov@r-pharm.com (M.S.); 5 Experimental Rheumatology, OvG University Magdeburg, 39120 Magdeburg, Germany; eugen.feist@med.ovgu.de; 6 Rheumatology and Clinical Immunology, HELIOS Fachklinik Vogelsang/Gommern, 39245 Gommern, Germany

**Keywords:** rheumatoid arthritis, antirheumatic agents, biological treatment, adalimumab, olokizumab, population PK/PD model

## Abstract

**Background/Objectives**: Olokizumab (OKZ) is a humanized monoclonal antibody targeting interleukin-6 (IL-6). OKZ demonstrated efficacy in subjects with active rheumatoid arthritis (RA) in randomized controlled trials versus adalimumab (ADLM) and placebo over 24 weeks, with sustained efficacy confirmed through 106 weeks in an open-label single-arm extension study (OLE). This analysis aimed to predict 1-year efficacy of OKZ 64 mg administered every two weeks (q2w) and every four weeks (q4w) compared with ADLM and placebo through virtual clinical trial simulations based on population pharmacokinetic/pharmacodynamic (PK/PD) models. **Methods**: Nonlinear mixed-effects models were developed using pooled data from phase III studies. Turnover models with a maximum inhibition I_max_-type relationship between OKZ concentrations and clinical scores were applied. Simulated clinical trials were performed to estimate 52-week response rates, defined as proportions of patients achieving low disease activity (DAS28-CRP ≤ 3.2) and remission (CDAI ≤ 2.8). The reliability of the simulated extrapolations was validated against published data. **Results**: Log-transformed C-reactive protein (CRP) baseline levels and region were identified as significant covariates, influencing the dynamics of RA activity scores. OKZ and ADLM showed comparable predicted efficacy profiles, with overlapping 95% prediction intervals, and both were superior to placebo. Model predictions showed strong agreement with published long-term data for ADLM and placebo and closely reproduced the actual 1-year response profiles from the OKZ OLE. **Conclusions**: The developed PK/PD models showed good agreement with observed long-term efficacy outcomes for OKZ, ADLM, and placebo in MTX-IR RA patients. Both OKZ dosing regimens sustained low disease activity and remission rates comparable with those for ADLM over 52 weeks, supporting their use as effective treatment options.

## 1. Introduction

Rheumatoid arthritis (RA) is a chronic inflammatory autoimmune disease characterized by synovial inflammation that leads to damage and dysfunction in the affected joints. If inadequately treated, this disease is one of the leading causes of functional disability among adults [1]. The main goal of RA treatment is to achieve remission or low disease activity (LDA) [2].

Interleukin-6 (IL-6) is a multifunctional cytokine that is involved in many fundamental processes, including bone metabolism, immune responses, and inflammation [3,4]. In RA, an immune-mediated inflammatory disease, IL-6 plays a pivotal role through two interconnected axes. IL-6 stimulates acute-phase protein production and systemic inflammation, driving fever, anemia, and systemic inflammation. Beyond its role as a downstream mediator of inflammation and of the hepatic acute-phase response, IL-6 shifts CD4+ T-cell differentiation toward Th17 cells while suppressing Treg generation [4,5,6,7]. This IL-6-driven Th17/Treg imbalance is reversible with IL-6 blockade and correlates with clinical improvement in RA [7,8,9]. Accordingly, biological agents targeting the IL-6 receptor (IL-6R) or the IL-6 signaling pathway demonstrated efficacy in RA patients.

Olokizumab (OKZ) is a humanized monoclonal antibody developed as an inhibitor of IL-6 that directly targets the IL-6 ligand rather than the IL-6R. The efficacy and safety of OKZ in combination with methotrexate (MTX) in subjects with moderately to severely active RA were demonstrated in the global phase III clinical trials program CREDO. The core CREDO program comprised three double-blind, randomized controlled trials (RCT), with controlled treatment periods of 24 weeks in RA patients with inadequate response to methotrexate (MTX-IR) (CREDO 1 and 2) or 16 weeks in RA patients refractory to tumor necrosis factor (TNF) inhibitors (TNFi-IR) (CREDO 3). In these pivotal studies, OKZ 64 mg administered either every two weeks (q2w) or every four weeks (q4w) demonstrated superiority over placebo and non-inferiority to adalimumab (ADLM) across multiple clinical outcomes [10,11,12]. After completion of the core trials, participants were eligible to continue receiving OKZ in an open-label extension (OLE) study, CREDO 4, that extended treatment for up to 2 years. Published long-term CREDO 4 results showed a sustained clinical response to OKZ over 106 weeks of treatment [13]. Although no formal statistical testing is generally recommended for OLE studies in RA [14,15], the absence of comparative data in uncontrolled OLE designs may complicate interpretation of results and reduce the certainty of the evidence generated [16,17,18]. A recognized methodological approach to address this issue is to contextualize single-arm trial data using external control groups derived from published outcomes, including historical RCTs or real-world data [17,18,19,20,21]. However, meaningful cross-trial comparisons remain challenging due to differences in trial design, patient selection, and outcome assessment between the CREDO core trials and available comparator literature.

Population pharmacokinetic/pharmacodynamic (PK/PD) modeling integrated with virtual clinical trial simulations represents a promising approach to mitigate these limitations [22,23]. By developing a mechanistic model using individual MTX-IR subject data, it is possible to generate long-term efficacy predictions for OKZ at both dosing regimens, as well as for placebo and ADLM. This unified modeling framework incorporates exposure–response relationships and a standardized set of patient covariates. The virtual trial paradigm enables estimation of clinically relevant endpoints including LDA defined as Disease Activity Score with 28-Joint Counts by C-Reactive Protein (DAS28-CRP ≤ 3.2), and remission, defined as Clinical Disease Activity Index (CDAI ≤ 2.8) [15,24]. The validity of model predictions could be confirmed by comparison with results from the CREDO 4 study and previously published long-term data for ADLM and placebo for similar patient populations [25,26,27,28].

Therefore, the aim of the current analysis was to develop population PK/PD models to assess comparative long-term (52-week) efficacy of OKZ 64 mg administered q2w and q4w, ADLM, and placebo in MTX-IR patients with moderately to severely active RA using virtual clinical trial simulations [22,23].

## 2. Materials and Methods

### 2.1. Data

#### 2.1.1. Data Sources and Patients

This post hoc analysis used the pooled individual-level data from two phase III randomized, double-blind, controlled clinical trials: CREDO 1 (NCT02760368) and CREDO 2 (NCT02760407); the trials’ design and results were published earlier [11,12]. Briefly, these studies included adults with moderately to severely active RA who had inadequately responded to methotrexate, i.e., MTX-IR patients. The dataset included 1314 patients with PK measurements who received OKZ (64 mg q2w or q4w), ADLM (40 mg q2w), or placebo, all in combination with MTX, over a 24-week period, and had at least one baseline and one post-baseline measurement of the pharmacodynamic (PD) endpoints: DAS28-CRP and CDAI (Table 1 and Table 2, Figures S1–S4, Tables S1 and S2).

#### 2.1.2. Published Long-Term Efficacy Outcomes

Different sources of published data for OKZ, ADLM, and placebo were used to compare these outcomes to the PK/PD model predictions. For OKZ, the published 1-year efficacy outcomes (DAS28-CRP and CDAI) from the open-label extension study CREDO 4 [13] were integrated using both non-responder imputation (NRI) and as-observed data.

To confirm predictions for long-term ADLM and placebo outcomes, a systematic literature review was conducted in accordance with the Preferred Reporting Items for Systematic Reviews and Meta-Analyses (PRISMA [29], Table S3).

### 2.2. Model Development and Evaluation

Population PK/PD analyses were conducted using nonlinear mixed-effects modeling to characterize the effect–time relationship, in accordance with US Food and Drug Administration guidance [30]. For both DAS28-CRP and CDAI, a sequential population PK parameters and data (PPP&D) approach was applied [31]. Base PK/PD models were developed using parsimonious structural models with inter-individual and residual variability. The covariance structure of inter-individual variability (IIV) was systematically evaluated and refined by adding or removing IIV terms or covariances based on parameter identifiability, relative standard error (RSE < 50%), changes in Akaike Information Criterion (AIC), and variance-based shrinkage (<51%).

Missing PD observations within the 24-week controlled period were not imputed for model estimation. Parameters were estimated by maximum likelihood on all available observations, so that each subject contributed according to the number of measurements actually recorded. This approach is valid under a missing-at-random assumption, in which the probability of a missing observation may depend on previously observed responses and covariates but not on the unobserved value itself. A joint model of the dropout process was not developed; the possibility of informative dropout therefore cannot be excluded and is addressed in Section Limitations. Non-responder imputation (NRI) was applied for the comparison of model predictions with published aggregate outcomes (Section 2.1.2).

Model adequacy was assessed using standard diagnostic plots and visual predictive checks (VPCs), comparing observed data with simulation-based prediction intervals following Bergstrand et al. [32], to evaluate central tendency and variability across the independent variable range.

Covariate model development followed a stepwise procedure starting from the base models, with evaluation of parameter precision, model stability, sensitivity, and biological and clinical interpretability of covariate effects. Model selection was guided by changes in objective function value (OFV), likelihood ratio tests, and AIC. Particularly, a stepwise algorithm of COSSAC (with the likelihood ratio test (LRT) and the threshold *p*-values of < 0.05 and < 0.001 for forward selection and backward elimination steps, respectively) [33] was applied to identify the final models in the current research.

Final models were verified through successful convergence, plausibility and precision of parameter estimates, and comprehensive diagnostics, including residual plots, random-effects distributions, correlations with covariates, and VPCs. The impact of covariates on PD parameters was summarized using forest plots for the corresponding simulation outcomes (Figures S17–S20). For continuous covariates, effects were evaluated at the 5th and 95th percentiles relative to the median, while categorical covariate effects were assessed relative to reference categories.

#### 2.2.1. Pharmacokinetic Model

For OKZ, a two-compartment PK model with linear clearance and first-order absorption after SC injection was selected for further PK/PD model development, consistent with the previously developed population PK model of OKZ [34]. That model was built on pooled data from the phase I-III OKZ studies in healthy volunteers and in subjects with RA (NCT01276119, NCT01009242, NCT01262794, NCT01296711, NCT01463059, NCT02760368, NCT02760407 and NCT02760433). Bioavailability after SC administration was fixed; a combined residual error model was applied, and concentrations below the limit of quantification were handled as censored data [34,35,36].

In the PK model, the random effects for clearance (CL) and central compartment volume (V1) were introduced. The log-transformed baseline covariate effects of serum albumin, high-density lipoprotein cholesterol, body weight, and alanine aminotransferase (logBALB, logBHDLC, logBWGT, and logBALT) were selected for CL, and logBALT as well as logBWGT were selected as the covariates for V1 [34] by means of a stepwise covariate search procedure. The PK model from [34] also included a correlation term between the random effects on CL and V1. As this correlation cannot be fixed for forward simulations in the modeling software, an equivalent parameterization was applied in which a scaled fraction of the V1 random effect was shared with CL, keeping adequate parameter identifiability (Table S6, Equations (S1) and (S2)).

#### 2.2.2. Pharmacodynamic Models

For DAS28-CRP score longitudinal model development, I_max_-type turnover models were tested to describe the relationship between OKZ concentration and DAS28-CRP changes [37]. To model a background effect of concomitant MTX therapy, the disease-modifying anti-rheumatic drug (DMARD) parameter was introduced to the model as an additional term to OKZ concentration:

(1) $$\frac{\mathrm{dR}}{\mathrm{dt}}=k_{\mathrm{out}}\cdot R_{0}\cdot \left(1-\frac{I_{\max}\cdot \left(\mathrm{DMARD}+{\mathrm{Conc}}_{\mathrm{OKZ}}\right)}{{\mathrm{IC}}_{50}+\left(\mathrm{DMARD}+{\mathrm{Conc}}_{\mathrm{OKZ}}\right)}\right)-k_{\mathrm{out}}\cdot R$$

where *R* is the level of the DAS28-CRP score, *R*_0_ is the baseline level of the DAS28-CRP score, *k_out_* is the DAS28-CRP score turnover rate, *I_max_* is the maximal inhibition level coefficient, *DMARD*—a term to model a background effect of concomitant therapy (methotrexate), *IC*_50_ is the concentration at 50% of maximum drug inhibitory effect, and *Conc*_OKZ_ is the concentration of OKZ.

In the case of ADLM with concomitant MTX treatment, the model was modified as follows:

(2) $$\frac{\mathrm{dR}}{\mathrm{dt}}=k_{\mathrm{out}}\cdot R_{0}\cdot \left(1-\frac{I_{\max}\cdot \mathrm{PADLM}}{{\mathrm{IC}}_{50}+\mathrm{PADLM}}\right)-k_{\mathrm{out}}\cdot R$$

The parameter *PADLM* was included to model the effect of ADLM with concomitant MTX treatment on the *R* variable (representing the DAS28-CRP score) time course. Thus, ADLM concentrations were not measured, and the PK was not modeled (the same as for MTX treatment). In Equation (2), *IC*_50_ is the parameter of OKZ treatment.

A similar approach (Equations (1) and (2)) was used for CDAI score longitudinal models with minor modifications: CDAI score was square root transformed for a more efficient parameter optimization procedure, while all generated graphical and table diagnostics incorporated back-transformed values. The responders’ proportion data were derived from DAS28-CRP or CDAI longitudinal model outcomes.

### 2.3. Virtual Clinical Trial Simulations and Comparison with Published Outcomes

The time course of DAS28-CRP and CDAI scores was predicted for OKZ 64 mg q2w and q4w, as well as ADLM and placebo, with concomitant MTX treatment from 0 up to 52 weeks. Multiple virtual populations of target size were generated, and PK/PD simulations (500 replicas per virtual study) were performed to estimate the uncertainty and variability in efficacy predictions. For simulated replicas of CREDO 4, a population size of *n* = 500 per arm was applied, consistent with the original study size; for all other studies (ADLM and placebo arms), exact population sizes as reported in the respective publications were used [13,25,26,27,28,38]. Due to the absence of individual data and covariates in ADLM and placebo published studies [25,26,27,28,38,39] the simulations were performed using the base PK/PD models. Baseline characteristics of the external comparator cohorts were broadly comparable with those of the CREDO 2 population, apart from the placebo (MTX) study [38], in which the mean baseline DAS28-CRP was higher than in CREDO 2 (Table S4).

The PK/PD predictions incorporated variability, residual error, and relevant covariate sets to provide prediction distributions or confidence/prediction intervals and check prediction interval (PI) overlap, as well as maintain comparability to published outcomes. To propagate estimation uncertainty into the predictions, replicates of the population parameters were sampled from the variance-covariance matrix of the parameter estimates of the final models rather than being fixed at their point estimates. For each replicate, virtual subjects were generated with random effects drawn from the estimated IIV distributions, and simulated observations incorporated residual error. The prediction intervals reported in Section 3 therefore jointly reflect parameter estimation uncertainty, inter-individual variability, and residual variability.

Population PK/PD modeling was performed in Monolix (Version 2024R1, Lixoft/Simulations Plus) using the Stochastic Approximation Expectation-Maximization (SAEM) with Importance Sampling (IMP) algorithm. Data preparation, post-processing, statistical analysis, and simulations were performed using R (version 4.2.3; the R Foundation for Statistical Computing).

## 3. Results

### 3.1. Disease Activity Score with 28-Joint Counts by C-Reactive Protein (DAS28-CRP) Population PK/PD Model

Given the initial data exploration and base model development, it was identified that the standard I_max_ model did not describe the slower component of the DAS28-CRP change observed in the data. Thus, an empirical dependence of the *I_max_* parameter on the time since treatment started was introduced into the DAS28-CRP (and further—in CDAI) base models:

(3) $$I_{\max,\;\mathrm{eff}}(t)=\frac{I_{\max}}{1+w_{1}\cdot \exp\left(-\beta_{1}\cdot t\right)}$$

In the model, the *I_max_*_,*eff*_ gradually increases from *I_max_*/(1 + *w*_1_) to *I_max_* value with time course, and this increase rate depends on parameter *β*_1_ (1/day).

**Figure 1:**
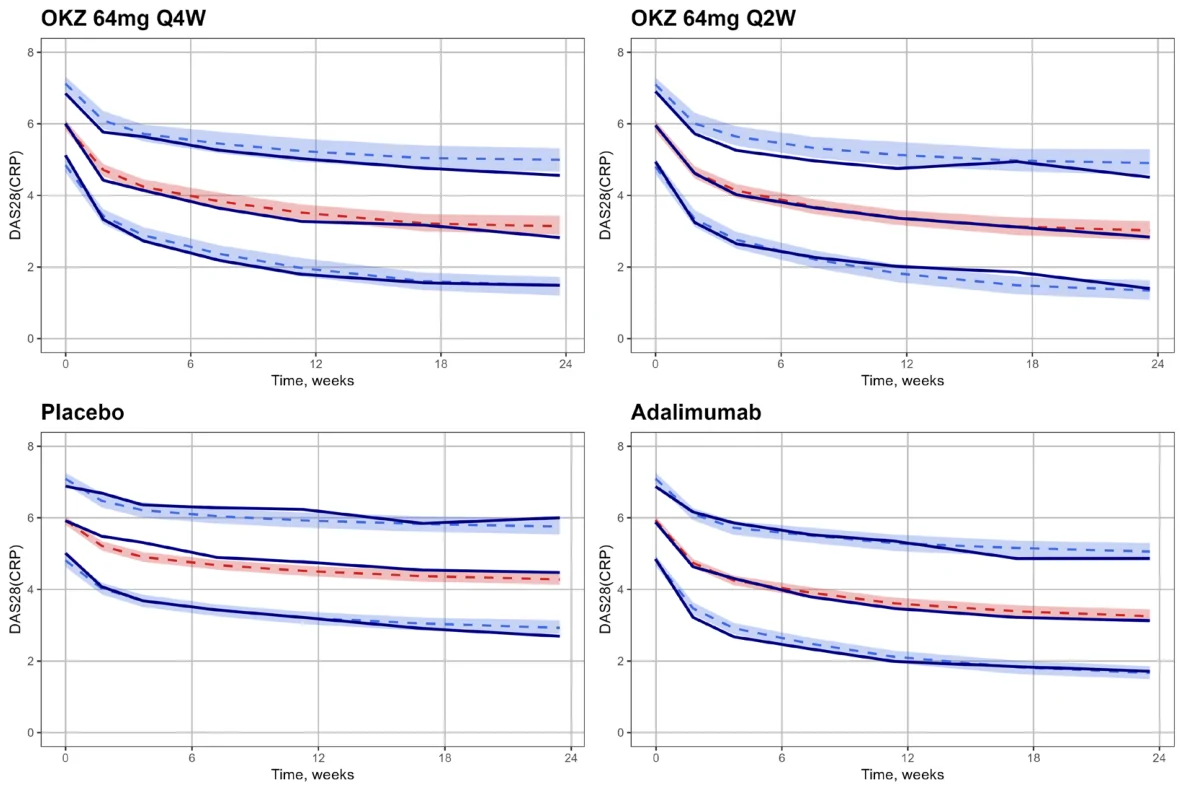
Final DAS28-CRP population PK/PD model visual predictive check. Solid dark-blue lines indicate the 5th, 50th, and 95th percentiles of experimental data (from bottom to top on the plot); dashed lines and corresponding ribbons indicate theoretical predictions of the percentiles with 95% confidence intervals (CI).

The selected base PK/PD model for DAS28-CRP incorporated random effects for *R_0_* and *I_max_*, a correlation term between the random effects, and an additive residual error (Table S7). No significant effect of ADLM treatment on *I_max_* time-dependent parameters was found. The final PK/PD model was obtained following a covariate search procedure that resulted in the inclusion of two covariates in the PD sub-model: log-transformed baseline CRP (logBCRP) for the baseline level of the DAS28-CRP score and the categorical covariate region (Latin America or not) on *I_max_*. The final model parameters were fully identifiable, and the model adequately described the data and its distribution by percentiles (Table 3, Table S8, Figure 1 and Figures S7, S11, S17 and S19).

### 3.2. Clinical Disease Activity Index (CDAI) Population PK/PD Model

For efficient parameter optimization during the base model development, in addition to the square root transformation of CDAI, the *PADLM* parameter was fixed to the fPADLM*IC_50_ value, where fPADLM was tested at 5, 10, or 20 to select a multiplier. Also, the ADLM treatment covariate coefficients were tested for the parameters *I_max_*, *w*_1_, and *β*_1_.

The selected base PK/PD model for CDAI was the I_max_ model with a time-dependent *I_max_* value (Equation (3)), random effects for *R_0_* and *I_max_*, a correlation term between the random effects, and a combined type of residual error. The parameter fPADLM = 10 was shown to be optimal (Table S9).

**Figure 2:**
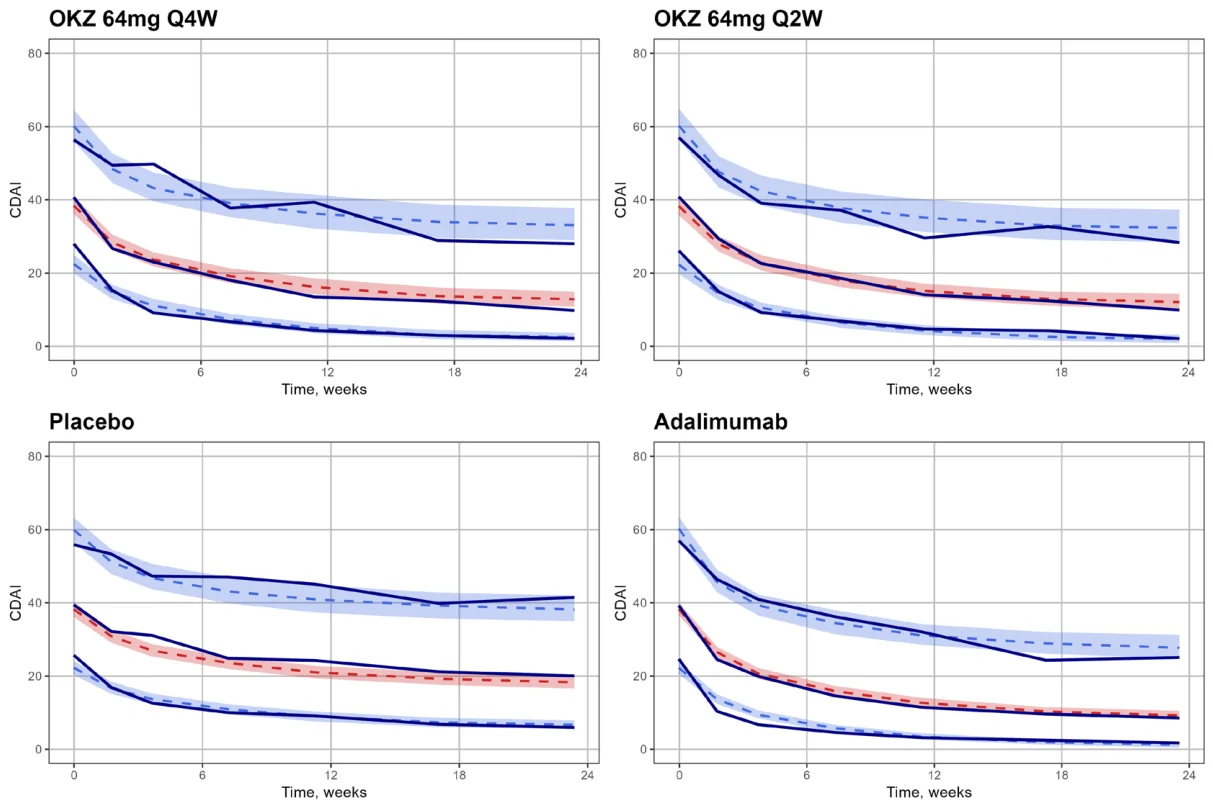
Final CDAI population PK/PD model visual predictive check. Solid lines indicate the 5th, 50th, and 95th percentiles of experimental data; dashed lines and corresponding ribbons indicate theoretical predictions of the percentiles with 95% CI.

Similar to DAS28-CRP, after the covariate search procedure, the final PK/PD model for CDAI included three covariate effects in the PD sub-model: baseline log-transformed CRP (logBCRP) for the baseline level of CDAI, the categorical covariate region (Latin America or not) for the *I_max_* parameter, and, as already identified during base model development, ADLM treatment (ADLMTRTc) for the *I_max_* parameter. The final model parameters were fully identifiable, and the model adequately described the data (Table 4 and Table S10, Figure 2 and Figures S12, S16, S18 and S20).

Similarly to the outcomes of DAS28-CRP, in the CDAI final PK/PD model, the covariate effect assessment at 24 weeks demonstrated that the highest effects were identified for logBCRP and the region (Latin America or not—Figures S18 and S20). These effects, however, were modest in magnitude, so that the statistically significant covariates identified for CDAI did not translate into a pharmacologically relevant impact on the simulated outcomes. The covariate assessments at different time points (12 and 52 weeks) showed similar results.

### 3.3. DAS28-CRP PK/PD Model Predicted Versus Published Data

**Figure 3:**
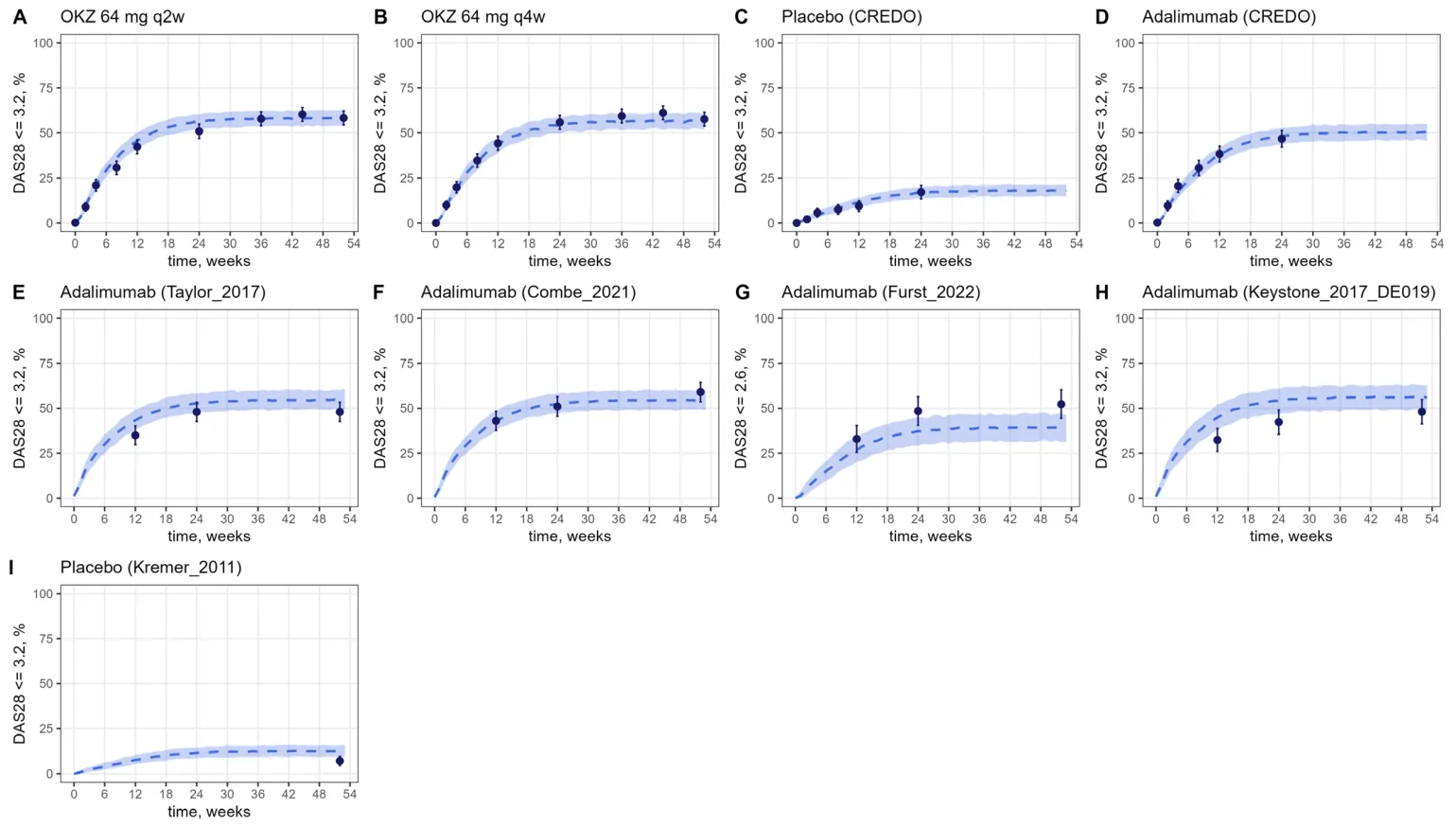
The DAS28-CRP population PK/PD model compared with published data: (**A**–**D**) final model predictions versus 52-week OKZ outcomes and 24-week ADLM and placebo outcomes from the CREDO studies; q2w every two weeks, q4w every four weeks. (**E**–**I**) Base model predictions versus published ADLM and placebo data [25,26,27,28,38]; panel (**G**) shows the percentage of patients with DAS28-CRP ≤ 2.6, whereas all remaining panels show DAS28-CRP ≤ 3.2. Dark blue dots indicate patients’ proportions with 95% CIs calculated using the NRI approach; the blue dashed line indicates the median prediction; the sky-blue ribbon indicates the 95% prediction interval.

The long-term DAS28-CRP ≤ 3.2 PK/PD final model predictions for OKZ 64 mg q2w and q4w in multiple virtual trials adequately reproduced the actual NRI-adjusted 1-year response profiles reported in the CREDO 4 study [13] (Figure 3A–D). The NRI approach was selected for this comparison due to being the most conservative approach, minimizing potential overestimation of treatment efficacy that may arise from the higher probability of dropout for non-responding subjects.

Similarly, for the proportion of MTX-IR subjects who achieved DAS28-CRP ≤ 3.2 over 12 months in the ADLM and placebo groups, the model predictions were consistent with the published aggregated long-term data (Figure 3E–I and Figure S5) collected by means of a systematic literature review (see the Supplementary Materials for more details). The uncertainty intervals provided for both the data and model-based simulations overlapped, indicating that the model-based extrapolations showed good agreement with the observed long-term ADLM and placebo outcomes (Figure 3E–I).

### 3.4. CDAI PK/PD Model Predicted Versus Published Data

**Figure 4:**
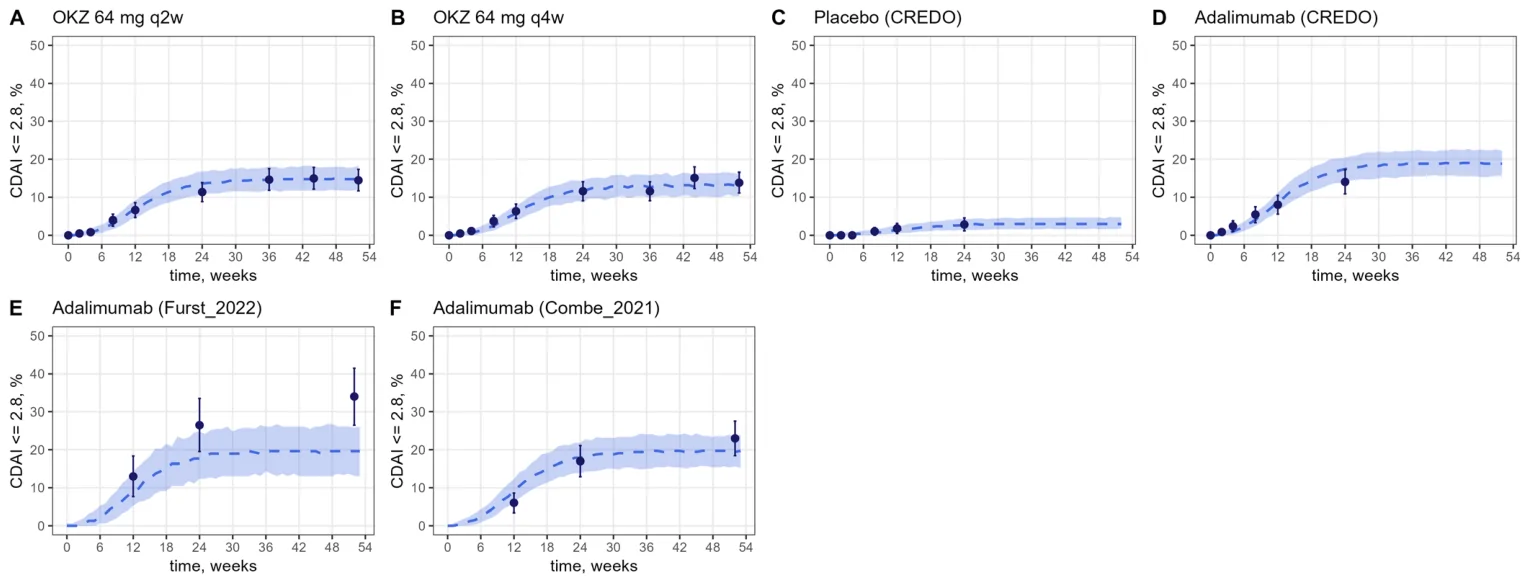
The CDAI population PK/PD model comparison to published data: (**A**–**D**) the final model comparison to 52-week OKZ data and 24-week ADLM and placebo data in the CREDO study; q2w every two weeks, q4w every four weeks. (**E**,**F**) The base model comparison to ADLM data [25,26,39]. Dark blue dots indicate patients’ proportions with 95% CIs calculated using NRI approaches; the blue dashed line indicates the median prediction; the sky-blue ribbon indicates the 95% prediction interval.

The long-term CDAI ≤ 2.8 PK/PD final model predictions for OKZ 64 mg q2w and q4w in multiple virtual trials closely reproduced the actual NRI-adjusted 1-year response profiles from the CREDO 4 study (Figure 4A–D). The NRI approach was selected for this comparison due to being the most conservative method, minimizing potential overestimation of treatment efficacy that may arise from the higher probability of dropout for non-responding subjects.

Similarly, for the proportion of MTX-IR subjects who achieved CDAI ≤ 2.8 over 12 months in the ADLM and placebo groups, the model predictions were consistent with the published long-term data (Figure 4E,F and Figure S6). The uncertainty intervals provided for both the data and model simulations overlapped, indicating that the model-based extrapolations showed good agreement with the observed long-term ADLM and placebo outcomes (Figure 4E,F).

### 3.5. DAS28-CRP and CDAI Response Rates Assessment by PK/PD Model Predictions

**Figure 5:**
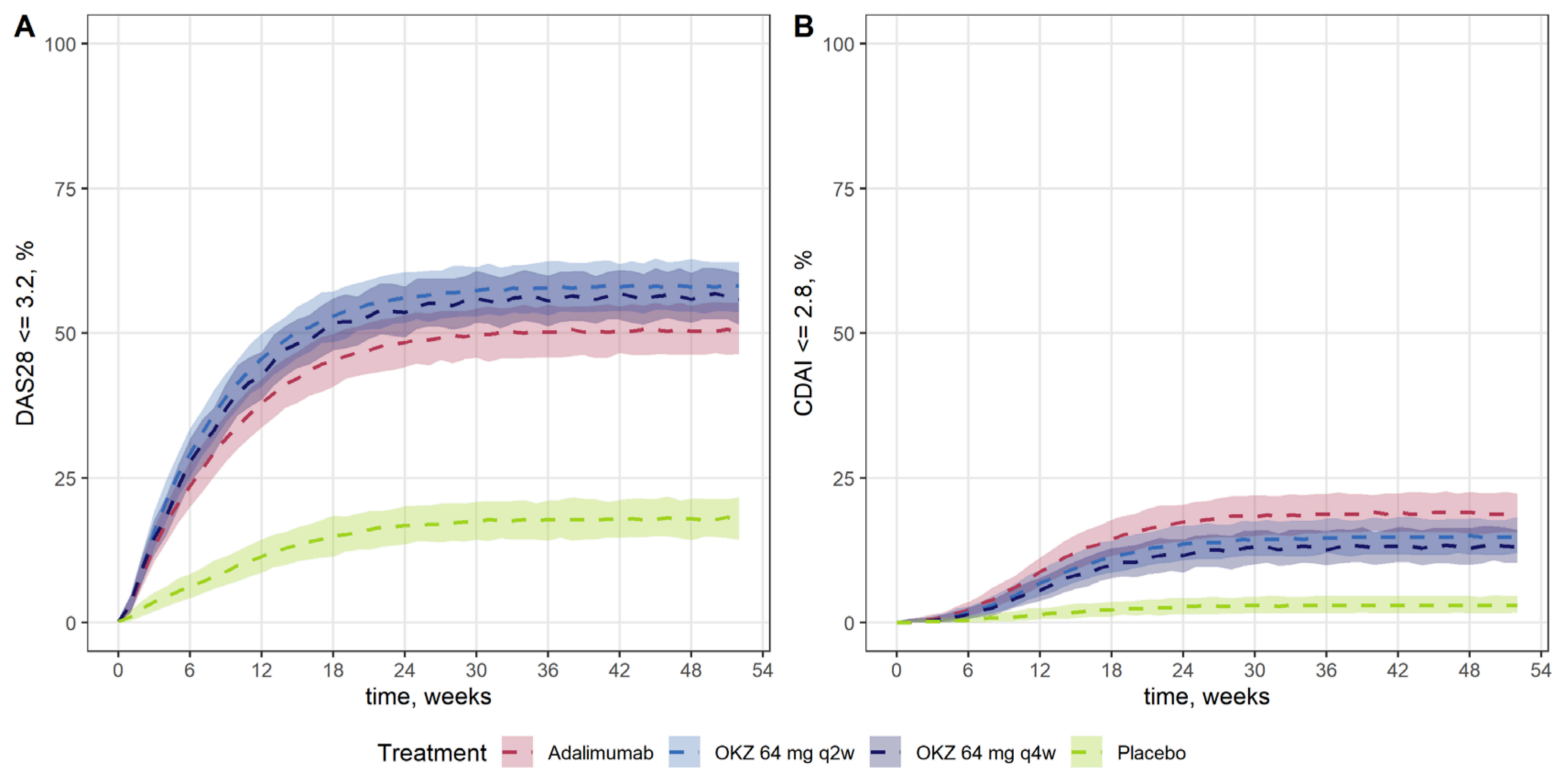
The DAS28-CRP (**A**) and CDAI (**B**) population PK/PD model predictions for OKZ 64 mg q2w, OKZ 64 mg q4w, ADLM 40 mg q2w, and placebo, each with concomitant MTX, over 52 weeks of treatment. Dashed lines indicate median predictions; ribbons indicate 95% prediction intervals.

Thus, the final PK/PD models for DAS28-CRP and CDAI score were used to generate simulations that demonstrated that the proportions of patients who achieved LDA or remission over a 12-month treatment period were similar for the OKZ 64 mg q2w, OKZ 64 mg q4w, and ADLM groups. The overlapping 95% PIs for OKZ and ADLM outcomes indicated comparable predicted efficacy for the active treatments, noting that no formal comparative statistical testing between the simulated arms was performed, while both OKZ and ADLM remained superior to placebo from week 24 to week 52, with no overlap in the 95% PIs (Figure 5, Table 5).

## 4. Discussion

MTX remains the anchor drug for RA, but a substantial proportion of patients respond inadequately and require escalation with a biologic DMARD. TNF inhibition with adalimumab and IL-6 pathway inhibition are both established biologic options; patients in whom TNF blockade is intolerable or insufficient may derive clinical benefit from switching to an alternative mechanism such as IL-6 inhibition [40].

Beyond its direct contribution to the acute-phase response and synovial inflammation, IL-6 plays an important position within the broader immunoregulatory network involved in RA. CD4+ T-cell responses comprise interacting Th1, Th2, Th17, and regulatory T-cell (Treg) pathways, among which the Th17/Treg axis is of particular relevance to RA. In an inflammatory cytokine environment, IL-6 can cooperate with TGF-β to favor Th17 differentiation while opposing TGF-β-mediated differentiation of FOXP3+ regulatory T cells, thereby shifting the balance toward inflammatory rather than regulatory immunity [5,6]. Consistent with this concept, IL-6 signaling blockade has been associated with a reduction in circulating Th17 cells, an increase in Tregs, and concomitant clinical improvement in patients with RA [7,8,9]. Thus, therapeutic neutralization of IL-6 may have biological consequences extending beyond suppression of the acute-phase response and may influence the balance between proinflammatory effector and regulatory immune pathways. These potential mechanisms were not evaluated in the present study but provide a broader immunologic context for interpreting the sustained clinical effects of IL-6 inhibition.

Olokizumab binds IL-6 at site 3 and prevents formation of the IL-6/IL-6R/gp130 signaling complex. In recent years, accumulating evidence has further expanded IL-6 signaling pathway pleiotropy: IL-6 can bind IL-11R and activate an IL-11R:gp130 complex associated with profibrotic signaling, whereas IL-6R can serve as a co-receptor for IL-30 (IL-27p28), mediating gp130-dependent, IL-10–linked anti-inflammatory effects other than canonical IL-6 signaling [41,42,43,44]. Thus, direct IL-6 neutralization and IL-6R blockade represent pharmacologically distinct approaches to inhibiting the IL-6 pathway. Although both strategies have demonstrated clinical efficacy in RA, the distinct epitope and signaling consequences of olokizumab provide a mechanistic rationale for its evaluation as an additional therapeutic option, including for patients with an inadequate response or intolerance to TNF inhibitors [45].

Because RA requires treatment over years rather than months, and the CREDO 4 study being uncontrolled, determining how the efficacy of OKZ compares with an established biological treatment beyond the controlled trial period is of direct clinical relevance. Following the recent judgment by regulatory authorities, interventional clinical trial data without concurrent control arms may be powered with the clinical data from external comparator groups (external control arms, ECAs) for further analysis [46,47]. Nevertheless, the use of such external data as a comparator group for single-arm OLE trials in chronic conditions such as immune-mediated inflammatory diseases may be challenging due to differences in patient cohort selection as well as variations in clinical trial design and conduct. One of the major challenges is the availability of high-quality individual-level data with comparable baseline characteristics suitable for the construction of ECAs.

In this work, we propose for the first time an alternative approach based on the development of longitudinal population PK/PD models of rheumatoid arthritis disease activity scores for different treatments derived from pivotal RCTs. The consistency between model predictions and outcomes observed in clinical trials provided evidence supporting the adequacy and validity of the model-based predictions. This framework enabled the comparison of the long-term efficacy of OKZ, ADLM, and placebo through multiple simulations of virtual clinical trials in randomized patient populations representative of the pooled CREDO1 and CREDO 2 populations.

According to this approach, pooled PK/PD data for MTX-IR subjects available from phase III studies CREDO 1 and CREDO 2 were utilized to develop population PK/PD models of OKZ, ADLM, and placebo treatments and perform simulations of long-term dynamics of DAS28-CRP and CDAI scores. The final PK/PD models for both DAS28-CRP and CDAI were turnover models with maximum inhibition I_max_-type relationships between OKZ concentration and DAS28-CRP and CDAI scores, respectively, in line with exposure-response models previously reported for other IL-6 pathway inhibitors such as tocilizumab [48]. The effects of placebo and ADLM treatment were considered in the same models. An empirical time-dependent increase in the maximum inhibitory effect was included in the models for capturing the slower, progressive improvement in disease activity scores observed in the data, suggesting that the full PD effect of IL-6 inhibition with OKZ and TNF inhibition with ADLM develops over several months. This time dependence is empirical and was introduced to describe the observed gradual component of the response; it does not represent a mechanistic model of any specific immunologic process. It should be noted that the parameters describing this empirical time dependence did not differ significantly across the investigated treatment groups and were consistently identified for both DAS28-CRP and CDAI score dynamics.

The final DAS28-CRP and CDAI models included one covariate on baseline PD score (baseline log-transformed CRP) and one covariate on *I_max_* (region: Latin America or not); the CDAI model additionally retained the effect of ADLM treatment on *I_max_*. Higher baseline inflammation logically correlates with higher starting disease activity, and higher response rates for all considered treatments were accordingly predicted by the model for patients with lower vs. higher baseline CRP. The regional effect on *I_max_*, by contrast, should be interpreted with caution. Although the CREDO studies were conducted as a standardized global clinical development program with harmonized eligibility criteria and disease activity assessments, residual subjectivity to which both DAS28-CRP and CDAI are sensitive cannot be excluded. For example, regional variation in patients’ perception of disease severity and pain, trust in medical interventions, local standards of care, and increased use of concomitant glucocorticoids have explained higher placebo response rates in Latin America documented in RA trials [49,50]. Consistently, a greater proportion of patients responded to both placebo and active treatment in Latin America compared with other regions in the CREDO studies. Furthermore, the region covariate on *I_max_* in the disease activity turnover model indicates differences in the predicted maximal PD effect across regions rather than in the exposure required to attain this effect; in other words, at comparable OKZ exposure and baseline characteristics, the model predicts different asymptotic levels of inhibition of the PD marker in different regions. Sensitivity analyses of placebo-corrected outcomes did not support dose adjustment based on baseline CRP or region. After placebo correction, their influence on the simulated OKZ and ADLM outcomes became negligible (Figures S19 and S20), indicating that it operates predominantly on the background response rather than on drug effect. Thus, both covariates have no pharmacological relevance for OKZ dose selection.

An additional consideration concerns the interpretation of the two modeled endpoints. IL-6 is a principal driver for hepatic CRP production, and IL-6-directed therapy therefore lowers CRP relatively directly through inhibition of the IL-6-acute-phase pathway. Because CRP is a component of DAS28-CRP, improvement in this index under IL-6 blockade could, in principle, reflect direct pharmacologic suppression of an acute-phase reactant rather than a change in underlying disease activity. Parallel modeling of CDAI, which comprises tender and swollen joint counts together with patient and physician global assessments and is CRP-independent, therefore represents an important strength of the present analysis. The consistent results obtained for a CRP-containing and CRP-independent disease activity index support the robustness and reliability of the study conclusions.

The model predictions for ADLM and placebo treatment groups were highly consistent with the published long-term data [25,26,27,28,38], indicating that the model-based extrapolations adequately reproduced the observed long-term ADLM and placebo outcomes. Also, it should be noted that patient characteristics as well as the published outcomes for ADLM and placebo expressed certain differences with respect to the ones from CREDO 2 as well as among each other (Table S4 and S5), that can be further addressed by an exhaustive meta-analysis. Additionally, the model predictions for OKZ 64 mg q2w and q4w closely reproduced the actual NRI-adjusted response profiles in the CREDO 4 study [13], further supporting the reliability of the simulated extrapolations.

### Limitations

Several limitations of this work should be acknowledged. Because the principal conclusions rest on PK/PD modeling and on extrapolation of controlled clinical trial observations to longer-term outcomes, they should be clearly distinguished from directly observed findings.

First, to reduce data variability and to ensure the maximal concordance between the baseline characteristics of CREDO patients and patients on ADLM from studies used for model validation, the models were developed exclusively in the MTX-IR population; their applicability to patients on monotherapy (without concomitant MTX) or to TNFi-IR subjects requires further investigation.

Second, response dynamics beyond the observed 6-month period of the CREDO 1 and CREDO 2 studies were not directly observed (e.g., change in the efficacy in the control group or in the discontinued patients’ proportion), so the 52-week results represent model-based extrapolation rather than measurement.

Third, the comparison with external ADLM and placebo outcomes, while comprehensive, relied on aggregate published results rather than individual patient-level data, which introduces a degree of uncertainty in the alignment; for the same reason, these simulations were performed with the base rather than the covariate models. The external comparator populations also differed from CREDO 2 and from one another in baseline characteristics and in missing-data handling (Tables S4 and S5), so residual heterogeneity between the compared cohorts cannot be excluded.

Fourth, unmeasured or residual confounding may affect the estimated treatment outcomes. As discussed above, the regional covariate may capture subjective differences; thus, the placebo response and considered trial outcomes in the current analysis cannot be differentiated.

Fifth, although parameters were estimated on all available observations under a missing-at-random assumption and the comparisons with observed long-term outcomes used the conservative NRI datasets, no joint model of the dropout process was assessed.

Sixth, although most of the structural parameters governing the PK/PD relationship were estimated with high precision (RSE ≤ 7.5%), several structural, covariate and comparator-effect parameters reached RSEs of up to approximately 25%, still well within the prespecified criterion of RSE < 50%. This estimation uncertainty is propagated into the reported prediction intervals, whereas parameters fixed for identifiability are not; additional clinical data would allow greater precision.

Seventh, the present analysis incorporated the data from late-phase clinical research; thus, Th17 cells, Tregs, IL-17, FOXP3, IL-10, or related immunological endpoints were not assessed, and it may be a matter of extended research by means of quantitative systems pharmacology.

Finally, the PK/PD analysis demonstrated quantitative relationships between exposure and response; however, these relationships should not be interpreted as direct evidence of the immunologic mechanisms responsible for sustained clinical benefit.

## 5. Conclusions

In conclusion, this population PK/PD modeling analysis successfully characterized the time course of DAS28-CRP and CDAI in MTX-IR RA patients treated with olokizumab, adalimumab, and placebo, enabling long-term predictions of low disease activity and remission rates that showed good agreement with independently observed outcomes. The analysis supported the sustained efficacy of both olokizumab dosing regimens (64 mg q2w and q4w) over 52 weeks and their comparable predicted efficacy relative to adalimumab as add-on therapy to MTX. Overall, the developed models provide a validated tool for predicting long-term outcomes and support the clinical use of olokizumab with a consistent dosing regimen across a wide patient demographic.

## Figures and Tables

**Table 1:** Clinical studies included in the population PK/PD analysis.

Clinical Study Identifier	Treatment Doses and Regimens ^1^	Population	Number of Subjects per Arm ^2^
NCT02760368 (CREDO 1; phase III)	OKZ 64 mg, SC q4w	258 RA patients with MTX-IR	63
	OKZ 64 mg, SC q2w		56
	Placebo		139
	24-week treatment duration		
NCT02760407 (CREDO 2; phase III)	OKZ 64 mg, SC q4w	1056 RA patients with MTX-IR	186
	OKZ 64 mg, SC q2w		171
	ADLM 40 mg, SC q2w		460
	Placebo		239
	24-week treatment duration		

ADLM: adalimumab, MTX-IR: inadequate response to methotrexate, OKZ: olokizumab, PK/PD: pharmacokinetic/pharmacodynamic, q2w: every two weeks, q4w: every four weeks, RA: rheumatoid arthritis, SC: subcutaneously. ^1^ For all treatment arms, background MTX therapy was administered. ^2^ Number of subjects to be included in the PK/PD modeling dataset.

**Table 2:** Demographic characteristics of the patients and covariates included in the dataset for the development of the DAS28-CRP and CDAI population PK/PD models.

Covariate or Characteristic	Value
Mean (SD) age, years	53.12 (12.04)
Mean (SD) baseline albumin, g/L	42.07 (3.17)
Mean (SD) baseline ALT, U/L	20.51 (13.27)
Mean (SD) baseline AST, U/L	19.89 (9.97)
Mean (SD) baseline bilirubin, µmol/L	6.23 (3.32)
Mean (SD) body mass index, kg/m^2^	28.21 (6.12)
Mean (SD) baseline CKD-EPI, mL/min/1.73 m^2^	99.71 (16.22)
Mean (SD) baseline creatinine clearance, mL/min	110.34 (28.06)
Mean (SD) baseline CRP, mg/L	20.05 (21.36)
Mean (SD) baseline HDL cholesterol, mg/dL	57.53 (16.43)
Mean (SD) baseline total protein, g/L	71.36 (5.10)
Mean (SD) body weight, kg	75.81 (17.92)
Mean (SD) height, cm	163.8 (8.74)
Ethnicity, *n* (%)	Hispanic or Latino: 427 (32.50%)
	Non-Hispanic or Latino: 887 (67.50%)
Region, *n* (%)	Latin America: 372 (28.30%)
	Others: 942 (71.7%)
Sex, *n* (%)	Male: 266 (20.20%)
	Female: 1048 (79.80%)

ALT: alanine aminotransferase; AST: aspartate aminotransferase; CKD-EPI: estimated glomerular filtration rate; CRP: C-reactive protein; HDL: high-density lipoprotein; SD: standard deviation.

**Table 3:** Final parameter estimates for the DAS28-CRP PK/PD model.

Parameter (Units)	Definition	Estimate	SE	RSE (%)
Fixed effects				
R_0_	DAS28-CRP baseline level	5.8900	0.0262	0.4450
β_R0_logBCRP_	logBCRP covariate coefficient on R_0_	0.0649	0.0037	5.6400
k_out_ (day^−1^)	Elimination rate constant for DAS28-CRP score in turnover equation	0.1410	0.0100	7.0800
I_max_	Maximal inhibition level coefficient	0.4950	0.0111	2.2300
β_Imax_RGN2=1_	Coefficient of the Region covariate on I_max_	0.3810	0.0644	16.900
IC_50_ (μg/mL)	OKZ concentration of the semi-maximal inhibition effect	0.5760	0.0263	4.5700
DMARD (μg/mL)	Additional term to model a background effect of methotrexate therapy	0.7200	0.0537	7.4600
PADLM (μg/mL)	Parameter of the ADLM with concomitant MTX treatment effect on DAS28	4.1800	0.7150	17.1000
w_1_	Weight of the I_max_ time-dependent part	0.9160	0.0184	2.0100
β_1_	Rate coefficient of I_max_ growth	0.0224	0.0013	5.6900
Random effects				
ω_R0_	R_0_ inter-individual variability	0.1000	0.0038	3.8300
ω_Imax_	I_max_ inter-individual variability	0.8580	0.0325	3.7900
Correlations				
Corr (R_0_, I_max_)	R_0_–I_max_ random effects correlation coefficient	−0.4210	0.0489	11.600
Residual error				
a	Additive error coefficient	0.6700	0.0061	0.9170

RSE: relative standard error, SE: standard error.

**Table 4:** Final parameter estimates for the CDAI PK/PD model.

Parameter (Units)	Definition	Estimate	SE	RSE (%)
Fixed effects				
R_0_	Square root of CDAI baseline level	6.2100	0.0318	0.5120
β_R0_logBCRP_	logBCRP covariate coefficient on R_0_	0.0204	0.0044	21.4000
k_out_ (day^−1^)	Elimination rate constant for CDAI score in turnover equation	0.0911	0.0059	6.4700
I_max_	Maximal inhibition level coefficient	0.4380	0.0146	3.3200
β_Imax_ADLMTRTc=1_	ADLM treatment covariate coefficient on I_max_	0.4180	0.0818	19.600
β_Imax_RGN2=1_	Coefficient of the Region covariate on I_max_	0.3470	0.0758	21.9000
IC_50_ (μg/mL)	Concentration of semi-maximal inhibition level	0.9920	0.0026	0.2600
DMARD (μg/mL)	Additional term to model a background effect of methotrexate therapy	2.0700	0.2850	13.8000
fPADLM	Parameter of the ADLM with concomitant MTX treatment effect on CDAI	10		
w_1_	Weight of the I_max_ time-dependent part	1.1800	0.0451	3.8200
β_1_	Rate coefficient of I_max_ growth	0.0244	0.0015	6.0500
Random effects				
ω_R0_	R_0_ inter-individual variability	0.1340	0.0044	3.2900
ω_Imax_	I_max_ inter-individual variability	1.0500	0.0379	3.6100
Correlations				
Corr (R_0_, I_max_)	R_0_–I_max_ random effects correlation coefficient	−0.2890	0.0448	15.5000
Residual error				
a	Additive term of the residual error	2.1400	0.1240	5.7800
b	Proportional residual error coefficient	0.2100	0.0068	3.2500

RSE: relative standard error, SE: standard error.

**Table 5:** Final PK/PD model predictions summary: proportions of patients who achieved LDA (DAS28-CRP ≤ 3.2) or remission (CDAI ≤ 2.8), median and 95% PI.

Treatment	DAS28-CRP ≤ 3.2 at Week 24 (PI 95%)	DAS28-CRP ≤ 3.2 at Week 52 (PI 95%)	CDAI ≤ 2.8 at Week 24 (PI 95%)	CDAI ≤ 2.8 at Week 52 (PI 95%)
OKZ 64 mg q2w + MTX	55.6 (51.0; 60.2)	57.4 (52.6; 62.2)	13.8 (11.1; 16.8)	15.4 (12.3; 18.7)
OKZ 64 mg q4w + MTX	53 (48.9; 57.2)	55 (50.6; 59.2)	11.8 (9.1; 14.8)	13.2 (10.7; 16.2)
ADLM 40 mg q2w + MTX	48 (43.6; 52.2)	49.8 (45.4; 53.8)	17.6 (14.4; 21.1)	19.4 (16.3; 22.8)
Placebo + MTX	17.2 (14.1; 20.6)	18.4 (15.4; 22.0)	2.8 (1.6; 4.4)	3.2(1.8; 5.0)

ADLM: adalimumab, CDAI: Clinical Disease Activity Index, DAS28-CRP: Disease Activity Score with 28-joint counts by C-reactive protein, MTX: methotrexate, OKZ: olokizumab, PI: prediction interval.

## Data Availability

All the relevant information was provided in the publication that describes the main study results (Nasonov et al. [11], Smolen et al. [12]). Upon a reasonable request to lemak@rpharm.ru, R-Pharm will provide access to individual de-identified participant data depending upon the nature of the request, the merit of the proposed research, the availability of the data, and its intended use.

## Supplementary Materials

The following supporting information can be downloaded at: https://www.mdpi.com/article/10.3390/jcm15197660/s1.

## Abbreviations

The following abbreviations are used in this manuscript:

ADLM	Adalimumab
AIC	Akaike information criterion
ALT	Alanine aminotransferase
AST	Aspartate aminotransferase
BMI	Body mass index
CDAI	Clinical Disease Activity Index
CI	Confidence interval
CKDEPI	Glomerular filtration rate estimated by Chronic Kidney Disease Epidemiology Collaboration equation
CL	Clearance
COSSAC	COnditional Sampling use for Stepwise Approach based on Correlation tests
CRP	C-Reactive protein
DAS28-CRP	Disease Activity Score with 28-Joint Counts by C-Reactive Protein
DMARD	Disease-modifying anti-rheumatic drug
ECA	External control arm
FOXP3	Forkhead box P3
HDL	High-density lipoprotein
IC_50_	OKZ concentration of semi-maximal inhibition level
IIV	Inter-individual variability
IL-6	Interleukin-6
IL-6R	Interleukin-6 receptor
IL-10	Interleukin-10
IL-17	Interleukin-17
I_max_	Maximal inhibition level coefficient
IMP	Importance sampling
LDA	Low disease activity
LOCF	Last observation carried forward
LRT	Likelihood ratio test
MTX	Methotrexate
MTX-IR	Inadequate response to methotrexate
NRI	Non-responder imputation
OFV	Objective function value
OKZ	Olokizumab
OLE	Open-label extension
PD	Pharmacodynamic(s)
PI	Prediction interval
PK	Pharmacokinetic(s)
PK/PD	Pharmacokinetic/pharmacodynamic
PPP&D	Population pharmacokinetic parameters and data
PRISMA	Preferred Reporting Items for Systematic Reviews and Meta-Analyses
q2w	Every two weeks
q4w	Every four weeks
RA	Rheumatoid arthritis
RCT	Randomized controlled trial
RSE	Relative standard error
SAEM	Stochastic approximation expectation-maximization
SC	Subcutaneous(ly)
SD	Standard deviation
SE	Standard error
TGF-β	Transforming growth factor-β
Th1	T helper type 1 cell
Th2	T helper type 2 cell
Th17	T helper type 17 cell
TNF	Tumor necrosis factor
TNFi-IR	Inadequate response to tumor necrosis factor inhibitors
Treg	Regulatory T cell
V1	Central compartment volume
VPC	Visual predictive check

## References

[1] Shi G., Liao X., Lin Z., Liu W., Luo X., Zhan H., Cai X. (2023). Estimation of the global prevalence, incidence, years lived with disability of rheumatoid arthritis in 2019 and forecasted incidence in 2040: Results from the Global Burden of Disease Study 2019. Clin. Rheumatol..

[2] Smolen J.S., Edwards C.J., Konzett V., Laskou F., Aletaha D., Caporali R., Dorner T., Hyrich K.L., Mateus E., Pope J.E. (2026). EULAR recommendations for the management of rheumatoid arthritis with synthetic and biologic disease-modifying antirheumatic drugs: 2025 update. Ann. Rheum. Dis..

[3] Bravo J., Heath J.K. (2000). Receptor recognition by gp130 cytokines. EMBO J..

[4] Jones S.A. (2005). Directing transition from innate to acquired immunity: Defining a role for IL-6. J. Immunol..

[5] Kimura A., Kishimoto T. (2010). IL-6: Regulator of Treg/Th17 balance. Eur. J. Immunol..

[6] Xie X., Liu Y., Li H. (2026). The Th17/Treg axis: A key to understanding and treating autoimmune disorders. Open Life Sci..

[7] Yoshida H., Magi M., Tamai H., Kikuchi J., Yoshimoto K., Otomo K., Matsumoto Y., Noguchi-Sasaki M., Takeuchi T., Kaneko Y. (2024). Effects of interleukin-6 signal inhibition on Treg subpopulations and association of Tregs with clinical outcomes in rheumatoid arthritis. Rheumatology.

[8] Pesce B., Soto L., Sabugo F., Wurmann P., Cuchacovich M., Lopez M.N., Sotelo P.H., Molina M.C., Aguillon J.C., Catalan D. (2013). Effect of interleukin-6 receptor blockade on the balance between regulatory T cells and T helper type 17 cells in rheumatoid arthritis patients. Clin. Exp. Immunol..

[9] Samson M., Audia S., Janikashvili N., Ciudad M., Trad M., Fraszczak J., Ornetti P., Maillefert J.F., Miossec P., Bonnotte B. (2012). Brief report: Inhibition of interleukin-6 function corrects Th17/Treg cell imbalance in patients with rheumatoid arthritis. Arthritis Rheum..

[10] Feist E., Fatenejad S., Grishin S., Korneva E., Luggen M.E., Nasonov E., Samsonov M., Smolen J.S., Fleischmann R.M. (2022). Olokizumab, a monoclonal antibody against interleukin-6, in combination with methotrexate in patients with rheumatoid arthritis inadequately controlled by tumour necrosis factor inhibitor therapy: Efficacy and safety results of a randomised controlled phase III study. Ann. Rheum. Dis..

[11] Nasonov E., Fatenejad S., Feist E., Ivanova M., Korneva E., Krechikova D.G., Maslyanskiy A.L., Samsonov M., Stoilov R., Zonova E.V. (2022). Olokizumab, a monoclonal antibody against interleukin 6, in combination with methotrexate in patients with rheumatoid arthritis inadequately controlled by methotrexate: Efficacy and safety results of a randomised controlled phase III study. Ann. Rheum. Dis..

[12] Smolen J.S., Feist E., Fatenejad S., Grishin S.A., Korneva E.V., Nasonov E.L., Samsonov M.Y., Fleischmann R.M., Group C. (2022). Olokizumab versus Placebo or Adalimumab in Rheumatoid Arthritis. N. Engl. J. Med..

[13] Feist E., Fleischmann R.M., Fatenejad S., Bukhanova D., Grishin S., Kuzkina S., Luggen M., Nasonov E., Samsonov M., Smolen J.S. (2024). Olokizumab plus methotrexate: Safety and efficacy over 106 weeks of treatment. Ann. Rheum. Dis..

[14] Buch M.H., Silva-Fernandez L., Carmona L., Aletaha D., Christensen R., Combe B., Emery P., Ferraccioli G., Guillemin F., Kvien T.K. (2015). Development of EULAR recommendations for the reporting of clinical trial extension studies in rheumatology. Ann. Rheum. Dis..

[15] European Medicines Agency: Committee for Medicinal Products for Human Use (CHMP) (2017). Guideline on Clinical Investigation of Medicinal Products for the Treatment of Rheumatoid Arthritis.

[16] Directorate-General for Health and Food Safety (2024). Methodological Guideline for Quantitative Evidence Synthesis: Direct and Indirect Comparisons.

[17] Wang C.Y., Berlin J.A., Gertz B., Davis K., Li J., Dreyer N.A., Zhou W., Seeger J.D., Santanello N., Winterstein A.G. (2022). Uncontrolled extensions of clinical trials and the use of external controls—Scoping opportunities and methods. Clin. Pharmacol. Ther..

[18] Seeger J.D., Davis K.J., Iannacone M.R., Zhou W., Dreyer N., Winterstein A.G., Santanello N., Gertz B., Berlin J.A. (2020). Methods for external control groups for single arm trials or long-term uncontrolled extensions to randomized clinical trials. Pharmacoepidemiol. Drug Saf..

[19] Lim J., Wang L., Best N., Liu J., Yuan J., Yong F., Zhang L., Walley R., Gosselin A., Roebling R. (2020). Reducing Patient Burden in Clinical Trials Through the Use of Historical Controls: Appropriate Selection of Historical Data to Minimize Risk of Bias. Ther. Innov. Regul. Sci..

[20] Rajagopal V., Jain A. Introduction to Synthetic Control Arm. Proceedings of the PharmaSUG.

[21] Zou K.H., Vigna C., Talwai A., Jain R., Galaznik A., Berger M.L., Li J.Z. (2024). The Next Horizon of Drug Development: External Control Arms and Innovative Tools to Enrich Clinical Trial Data. Ther. Innov. Regul. Sci..

[22] Tan J.M., Upton R.N., Foster D.J.R., Proudman S.M., Dhir V., Wiese M.D. (2024). Pharmacokinetic-pharmacodynamic modelling and simulation of methotrexate dosing in patients with rheumatoid arthritis. Br. J. Clin. Pharmacol..

[23] Craig M., Gevertz J.L., Kareva I., Wilkie K.P. (2023). A practical guide for the generation of model-based virtual clinical trials. Front. Syst. Biol..

[24] U.S. Food and Drug Administration (2013). Rheumatoid Arthritis: Developing Drug Products for Treatment. Guidance for Industry.

[25] Combe B., Kivitz A., Tanaka Y., van der Heijde D., Simon J.A., Baraf H.S.B., Kumar U., Matzkies F., Bartok B., Ye L. (2021). Filgotinib versus placebo or adalimumab in patients with rheumatoid arthritis and inadequate response to methotrexate: A phase III randomised clinical trial. Ann. Rheum. Dis..

[26] Furst D.E., Jaworski J., Wojciechowski R., Wiland P., Dudek A., Krogulec M., Jeka S., Zielinska A., Trefler J., Bartnicka-Maslowska K. (2022). Efficacy and safety of switching from reference adalimumab to CT-P17 (100 mg/ml): 52-week randomized, double-blind study in rheumatoid arthritis. Rheumatology.

[27] Keystone E.C., Breedveld F.C., van der Heijde D., van Vollenhoven R.F., Emery P., Smolen J.S., Sainsbury I., Florentinus S., Kupper H., Chen K. (2017). Achieving comprehensive disease control in patients with early and established rheumatoid arthritis treated with adalimumab plus methotrexate versus methotrexate alone. RMD Open.

[28] Taylor P.C., Keystone E.C., van der Heijde D., Weinblatt M.E., Del Carmen Morales L., Reyes Gonzaga J., Yakushin S., Ishii T., Emoto K., Beattie S. (2017). Baricitinib versus Placebo or Adalimumab in Rheumatoid Arthritis. N. Engl. J. Med..

[29] Page M.J., McKenzie J.E., Bossuyt P.M., Boutron I., Hoffmann T.C., Mulrow C.D., Shamseer L., Tetzlaff J.M., Akl E.A., Brennan S.E. (2021). The PRISMA 2020 statement: An updated guideline for reporting systematic reviews. BMJ.

[30] U.S. Food and Drug Administration (2022). Population Pharmacokinetics. Guidance for Industry.

[31] Zhang L., Beal S.L., Sheiner L.B. (2003). Simultaneous vs. sequential analysis for population PK/PD data I: Best-case performance. J. Pharmacokinet. Pharmacodyn..

[32] Bergstrand M., Hooker A.C., Wallin J.E., Karlsson M.O. (2011). Prediction-corrected visual predictive checks for diagnosing nonlinear mixed-effects models. AAPS J..

[33] Ayral G., Si Abdallah J.F., Magnard C., Chauvin J. (2021). A novel method based on unbiased correlations tests for covariate selection in nonlinear mixed effects models: The COSSAC approach. CPT Pharmacomet. Syst. Pharmacol..

[34] Zhudenkov K., Azarov I., Kosinsky Y., Peskov K., Grishin S., Egorova A., Lemak M., Samsonov M. Population Pharmacokinetics of Olokizumab in Healthy Volunteers and Rheumatoid Arthritis Subjects. https://www.page-meeting.org/wp-content/uploads/pdf_abstracts/2026/24a8b477251041a1af811c25981be7fe.pdf.

[35] How Monolix Handles Censored Data. https://monolix.lixoft.com/censoreddata/.

[36] Irby D.J., Ibrahim M.E., Dauki A.M., Badawi M.A., Illamola S.M., Chen M., Wang Y., Liu X., Phelps M.A., Mould D.R. (2021). Approaches to handling missing or “problematic” pharmacology data: Pharmacokinetics. CPT Pharmacomet. Syst. Pharmacol..

[37] Dayneka N.L., Garg V., Jusko W.J. (1993). Comparison of four basic models of indirect pharmacodynamic responses. J. Pharmacokinet. Biopharm..

[38] Kremer J.M., Blanco R., Brzosko M., Burgos-Vargas R., Halland A.M., Vernon E., Ambs P., Fleischmann R. (2011). Tocilizumab inhibits structural joint damage in rheumatoid arthritis patients with inadequate responses to methotrexate: Results from the double-blind treatment phase of a randomized placebo-controlled trial of tocilizumab safety and prevention of structural joint damage at one year. Arthritis Rheum..

[39] Kay J., Jaworski J., Wojciechowski R., Wiland P., Dudek A., Krogulec M., Jeka S., Zielinska A., Trefler J., Bartnicka-Maslowska K. (2021). Efficacy and safety of biosimilar CT-P17 versus reference adalimumab in subjects with rheumatoid arthritis: 24-week results from a randomized study. Arthritis Res. Ther..

[40] Scirica B.M., Cannon C.P., Sabatine M.S., Jarolim P., Sloane S., Rifai N., Braunwald E., Morrow D.A., Investigators P.I.T. (2009). Concentrations of C-reactive protein and B-type natriuretic peptide 30 days after acute coronary syndromes independently predict hospitalization for heart failure and cardiovascular death. Clin. Chem..

[41] Garbers C., Spudy B., Aparicio-Siegmund S., Waetzig G.H., Sommer J., Holscher C., Rose-John S., Grotzinger J., Lorenzen I., Scheller J. (2013). An interleukin-6 receptor-dependent molecular switch mediates signal transduction of the IL-27 cytokine subunit p28 (IL-30) via a gp130 protein receptor homodimer. J. Biol. Chem..

[42] Rose-John S., Jones S.A. (2025). More and more pleiotropy within the IL-6 family of cytokines. FEBS J..

[43] Weitz H.T., Ettich J., Rafii P., Wittich C., Schultz L., Frank N.C., Heise D., Krusche M., Lokau J., Garbers C. (2025). Interleukin-11 receptor is an alternative alpha-receptor for interleukin-6 and the chimeric cytokine IC7. FEBS J..

[44] Yan J., Mitra A., Hu J., Cutrera J.J., Xia X., Doetschman T., Gagea M., Mishra L., Li S. (2016). Interleukin-30 (IL27p28) alleviates experimental sepsis by modulating cytokine profile in NKT cells. J. Hepatol..

[45] Avci A.B., Feist E., Burmester G.R. (2024). Targeting IL-6 or IL-6 Receptor in Rheumatoid Arthritis: What Have We Learned?. BioDrugs.

[46] European Medicines Agency (2023). Real-World Evidence Framework to Support EU Regulatory Decision-Making: Report on the Experience Gained with Regulator-Led Studies from September 2021 to February 2023.

[47] U.S. Food and Drug Administration (2023). Considerations for the Design and Conduct of Externally Controlled Trials for Drug and Biological Products Guidance for Industry.

[48] Bastida C., Soy D., Ruiz-Esquide V., Sanmarti R., Huitema A.D.R. (2019). Exposure-response modeling of tocilizumab in rheumatoid arthritis using continuous composite measures and their individual components. Br. J. Clin. Pharmacol..

[49] Keebler D., Teng E., Chia J., Galanter J., Peake J., Tuckwell K. (2020). Regional variations in adverse event reporting rates and ACR responses in placebo/standard-of-care arms of rheumatoid arthritis trials. Rheumatology.

[50] Sokka T., Hetland M.L., Makinen H., Kautiainen H., Horslev-Petersen K., Luukkainen R.K., Combe B., Badsha H., Drosos A.A., Devlin J. (2008). Remission and rheumatoid arthritis: Data on patients receiving usual care in twenty-four countries. Arthritis Rheum..

