# Supplementary material for: Population PK/PD Modeling and Simulation to Predict the Comparative Long-Term Efficacy of Olokizumab in Rheumatoid Arthritis Patients
Source: J Clin Med. 2026 Oct 2;15(19):7660. doi: 10.3390/jcm15197660 (PMC13665878; doi:10.3390/jcm15197660)
Supplement: Supplementary file 1 [file jcm-15-07660-s001.zip › jcm-4490398-supplementary.pdf]

## Clinical data used for model development

For the development of population PK models, the pooled data from phase I-III OKZ clinical studies involving healthy volunteers and subjects with RA were used (NCT01276119, NCT01009242, NCT01262794, NCT01296711, NCT01463059, NCT02760368, NCT02760407, NCT02760433). For current PK/PD analysis, the model development was performed on a subset of the full dataset extracted from CREDO 1 (N=258) and CREDO 2 (N=1056) studies [7,8] encompassing subjects with the inadequate response to MTX (MTX-IR) (Table S1). The details about the criteria of the subjects' exclusion from the full dataset were added below to Supplementary document

*Table S1. Summary of studies included in the modeling dataset and study characteristics*

| Study Number | Study Description | Doses (N)$^{\#}$ | PD Variables included in the current analysis | PD Assessment Times |
|---|---|---|---|---|
| CL04041022 (CREDO 1, Phase III) NCT02760368 | A Randomized, Double-Blind, Parallel-Group, Placebo-Controlled, Multicenter Phase III Study of the Efficacy and Safety of Olokizumab in Subjects with Moderately to Severely Active Rheumatoid Arthritis Inadequately Controlled by Methotrexate Therapy | 64 mg q4w (63)<br>64 mg q2w (56)<br>PBO (139)<br><br>Total N = 258 | DAS28-CRP<br>CDAI | 0, 2, 4, 8, 12, 20, 24 weeks |
| CL04041023 (CREDO 2, Phase III) NCT02760407 | A Randomized, Double-Blind, Parallel-Group, Placebo- and Active-Controlled, Multicenter Phase III Study of the Efficacy and Safety of Olokizumab in Subjects with Moderately to Severely Active Rheumatoid Arthritis Inadequately Controlled by Methotrexate Therapy | 64 mg q4w (186)<br>64 mg q2w (171)<br>ADLM (460)<br>PBO (239)<br><br>Total N = 1056 | DAS28-CRP<br>CDAI | 0, 2, 4, 8, 12, 20, 24 weeks |

$^{\#}$ - Number of subjects to be included into PK/PD modeling dataset

Population for PK/PD analysis included subjects with RA from OKZ PK population and subjects from ADLM and placebo (PBO) groups who had at least one postbaseline measurement of PD variables (and must have baseline PD measurements): DAS28-CRP, CDAI. The modeling dataset finally included 227 OKZ 64 mg q2w, 249 OKZ 64 mg q4w, 378 PBO and 460 ADLM treated subjects.

The PK/PD modeling data file contains all essential information, such as subject identification, dosing details, sample collection time, baseline (and longitudinal if applicable) demographics, physiological and laboratory attributes. The data includes OKZ PK measurements, DAS28-CRP and CDAI scores and information pertaining to PD responses.

## PD data exploration

The aim was to generate accurate model predictions of DAS28-CRP and CDAI values overtime for MTX-IR subgroup and to compare these predictions with the published outcomes for ADLM and PBO, as the majority of the previously published long-term efficacy data were collected from the MTX-IR RA populations [21,22,24,28,35].

DAS28-CRP and CDAI dynamics generally were comparable in OKZ-treated groups across core studies (Figure S1-S2). DAS28-CRP and CDAI dynamics in ADLM-treated cohort in CREDO 2 study were comparable with OKZ-treated cohort and differ significantly from those in PBO group.

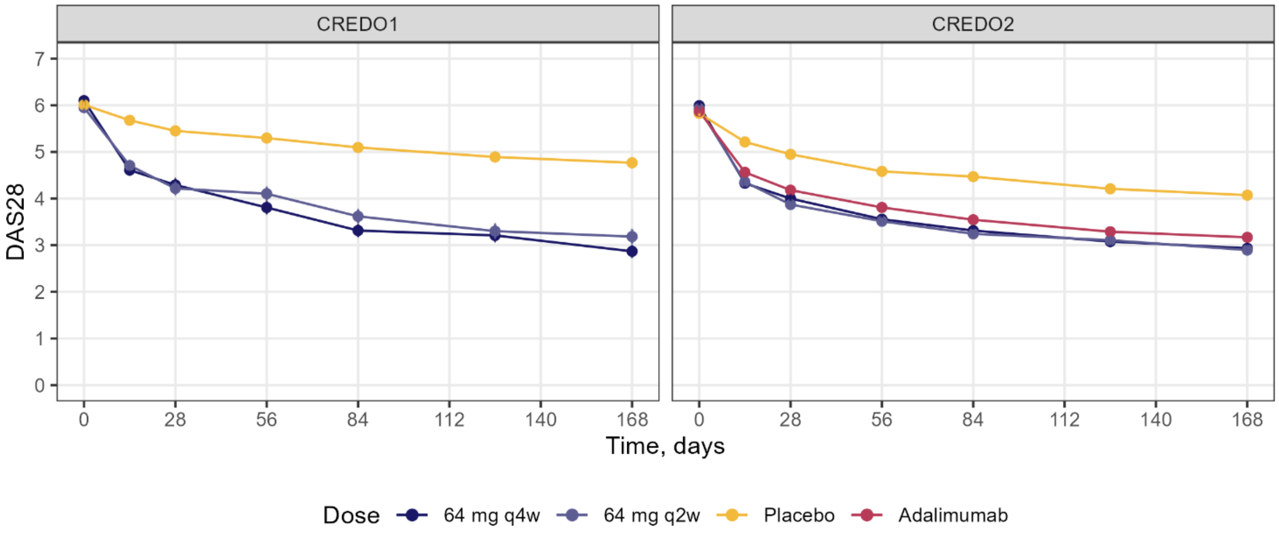


*Figure S1. Selected for PK/PD analysis clinical study outcomes: DAS28-CRP. Average values as well as SEs are provided for nominal observation times.*

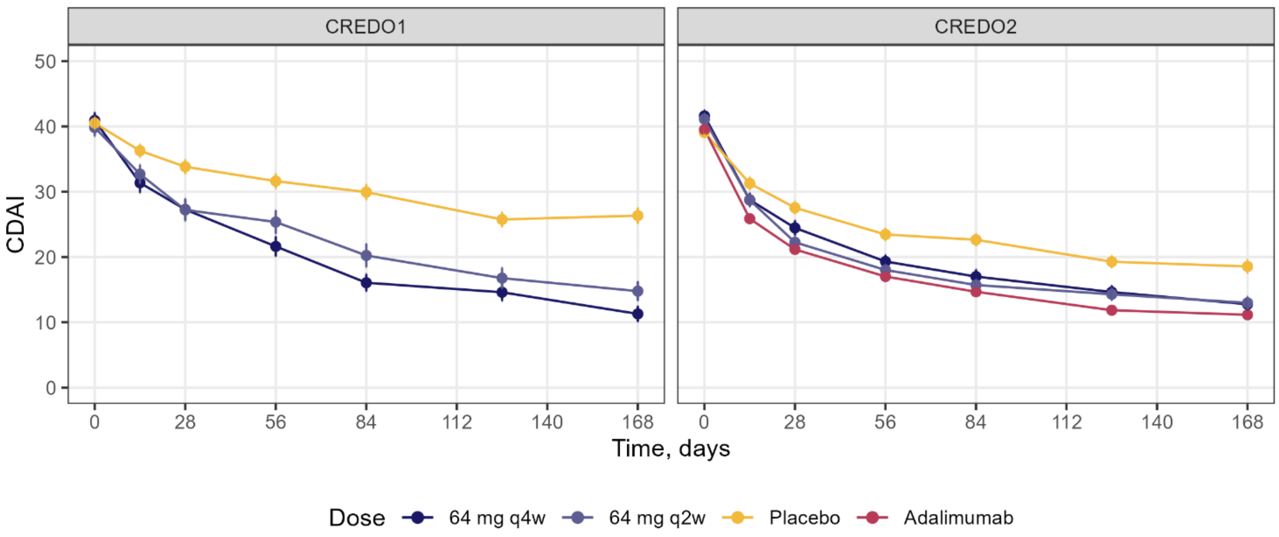


*Figure S2. Selected for PK/PD analysis clinical study outcomes: CDAI. Average values as well as SEs are provided for nominal observation times.*

## Description of covariates

Table S2 presents summary statistics for patient characteristics and covariates used in model development. All the considered continuous covariates were time-invariant in the studies or presented at baseline. The covariates are in the physiologically plausible values and correspond to inclusion-exclusion criteria of the studies [7,8]. Most of subjects in clinical development of OKZ for MTX-IR RA were from population of Eastern Europe (54.4%), Latin America (28.3%), and United States or Western Europe (16.0%). 84.0% of the subjects were White, while other races included American Indian or Alaska Native (4.5%), Asian (4.4%), Black or African American (1.7%) and other races (5.4%).

*Table S2. Summary of covariates in CREDO 1 and CREDO 2 studies.*

| **Study** | **CREDO 1** (n = 258)<br>Mean ± SD<br>Median<br>(Min, Max) | **CREDO 2** (n = 1056)<br>Mean ± SD<br>Median<br>(Min, Max) | **Total** (n = 1314)<br>Mean ± SD<br>Median<br>(Min, Max) |
|---|---|---|---|
| **AGE (years)** | 51.41 ± 11.13<br>52 (23, 80) | 53.54 ± 12.22<br>54 (19, 88) | 53.12 ± 12.04<br>54 (19, 88) |
| **BALB (g/L)** | 42.04 ± 3.06<br>42 (34, 52) | 42.07 ± 3.19<br>42 (33, 54) | 42.07 ± 3.17<br>42 (33, 54) |
| **BALT (U/L)** | 20.1 ± 16.45<br>15 (4, 179) | 20.61 ± 12.37<br>17 (5, 118) | 20.51 ± 13.27<br>17 (4, 179) |
| **BAST (U/L)** | 19.63 ± 11.57<br>17 (7, 110) | 19.96 ± 9.54<br>18 (2, 122) | 19.89 ± 9.97<br>18 (2, 122) |
| **BBILI (µmol/L)** | 6.27 ± 3.38<br>6 (1, 22) | 6.22 ± 3.31<br>5 (1, 26) | 6.23 ± 3.32<br>5 (1, 26) |
| **BBMI (kg/m$^2$)** | 26.71 ± 4.83<br>26.09 (17.46, 45.52) | 28.58 ± 6.34<br>27.54 (15.62, 62.22) | 28.21 ± 6.12<br>27.27 (15.62, 62.22) |
| **BCKDEPI (mL/min/1.73 m$^2$)** | 99.69 ± 15.56<br>101.33 (50.35, 140.08) | 99.71 ± 16.38<br>102 (38, 145.53) | 99.71 ± 16.22<br>101.95 (38, 145.53) |
| **BCRCL (mL/min)** | 108.68 ± 26.28<br>107.56 (42.78, 150) | 110.75 ± 28.48<br>109.83 (43.71, 150) | 110.34 ± 28.06<br>109.07 (42.78, 150) |
| **BCRP (mg/L)** | 25.33 ± 26.45<br>16 (0.3, 221) | 18.76 ± 19.72<br>12 (0.3, 175) | 20.05 ± 21.36<br>12.5 (0.3, 221) |

| Study | **CREDO 1** (n = 258)<br>Mean ± SD<br>Median<br>(Min, Max) | **CREDO 2** (n = 1056)<br>Mean ± SD<br>Median<br>(Min, Max) | **Total** (n = 1314)<br>Mean ± SD<br>Median<br>(Min, Max) |
|---|---|---|---|
| **BHDLC (mg/dL)** | 58.32 ± 16.18<br>57.53 (25.1, 142.08) | 57.34 ± 16.49<br>54.83 (20.08, 193.82) | 57.53 ± 16.43<br>54.83 (20.08, 193.82) |
| **BTOTP (g/L)** | 72.29 ± 4.71<br>72 (62, 88) | 71.14 ± 5.17<br>71 (31, 97) | 71.36 ± 5.1<br>71 (31, 97) |
| **BWGT (kg)** | 73.27 ± 14.53<br>71 (43, 125) | 76.44 ± 18.61<br>74 (40, 173.7) | 75.81 ± 17.92<br>73 (40, 173.7) |
| **HGT (cm)** | 165.55 ± 7.6<br>165 (146, 192) | 163.37 ± 8.95<br>162.5 (134.6, 193) | 163.8 ± 8.74<br>163 (134.6, 193) |
| **ETHN** | Non-Hispanic or Latino: 258 (100.0%) | Hispanic or Latino: 427 (40.4%)<br>Non-Hispanic or Latino: 629 (59.6%) | Hispanic or Latino: 427 (32.5%)<br>Non-Hispanic or Latino: 887 (67.5%) |
| **RACE** | White: 257 (99.6%)<br>Asian: 1 (0.4%) | White: 847 (80.2%)<br>American Indian or Alaska Native: 59 (5.6%)<br>Black: 21 (2.0%)<br>Other: 71 (6.7%)<br>Asian: 58 (5.5%) | White: 1104 (84.0%)<br>American Indian or Alaska Native: 59 (4.5%)<br>Black: 21 (1.6%)<br>Other: 71 (5.4%)<br>Asian: 59 (4.4%) |
| **Region** | Eastern Europe: 258 (100.0%) | United States: 210* (19.9%)<br>Eastern Europe: 457 (43.3%)<br>Latin America: 372 (35.2%)<br>Asia: 17 (1.6%) | United States: 210* (16.0%)<br>Eastern Europe: 715 (54.4%)<br>Latin America: 372 (28.3%)<br>Asia: 17 (1.3%) |
| **RGN2** | Other: 258 (100.0%) | Other: 684 (64.8%)<br>Latin America: 372 (35.2%) | Other: 942 (71.7%)<br>Latin America: 372 (28.3%) |
| **SEX** | Male: 42 (16.3%)<br>Female: 216 (83.7%) | Male: 224 (21.2%)<br>Female: 832 (78.8%) | Male: 266 (20.2%)<br>Female: 1048 (79.8%) |
| **Therapy Line** | RA Patients Inadequately Controlled by MTX: 258 (100.0%) | RA Patients Inadequately Controlled by MTX: 1056 (100.0%) | RA Patients Inadequately Controlled by MTX: 1314 (100.0%) |

*Including 24 subjects from Western Europe

Distributions of most of the considered continuous covariates were far from normal (Figure S3A). Non-normality of covariate distribution may become an issue for model convergence, especially cases of sophisticated PK/PD models. To overcome this, the continuous covariates were logarithmically transformed. After the log-transformation of the covariates their distribution became closer to normal, except creatinine clearance (logBCRCL) due to its maximum calculated values truncated at 150 mL/min (Figure S3B):

**A**

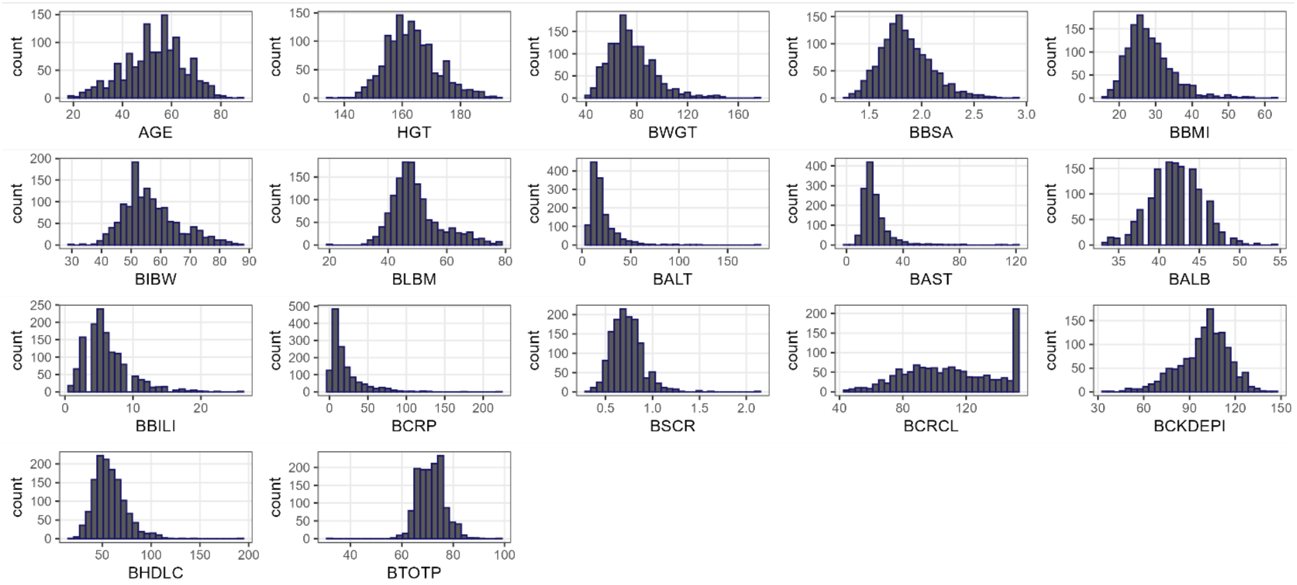


**B**

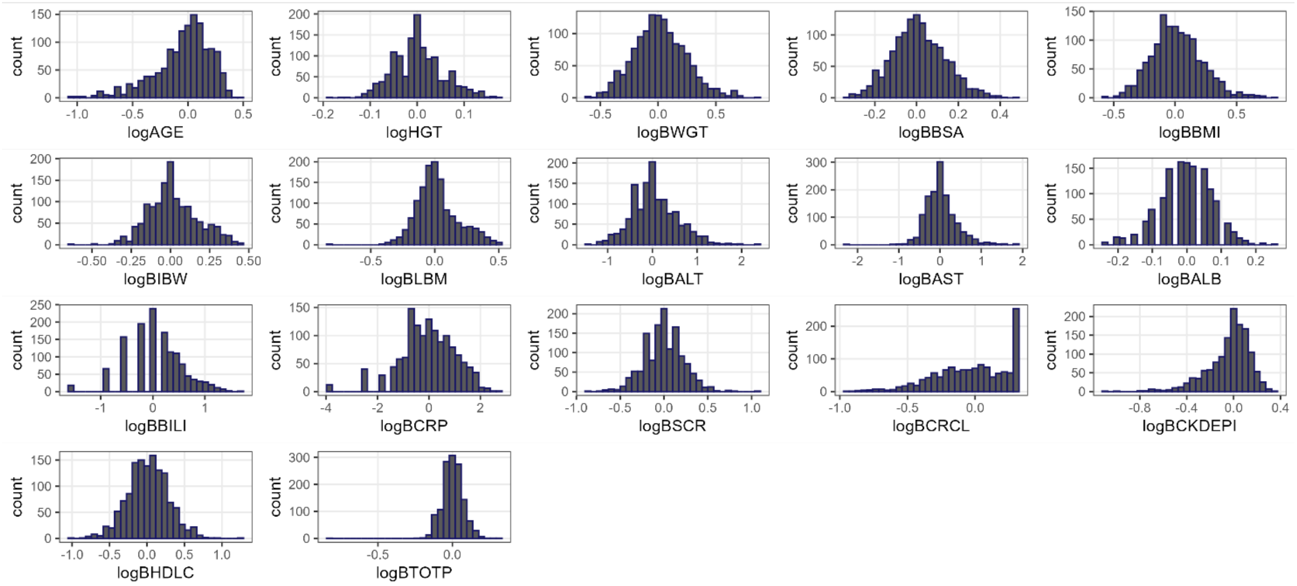


*Figure S3. Distribution of baseline covariates in CREDO 1 and CREDO 2 studies. A – standard values. B – logarithmic transformation of covariates.*

According to correlation plot several covariates correlate with each other (Pearson correlation coefficient was assessed): log-transformed height (logHGT), baseline log-transformed body mass index (logBBMI), baseline log-transformed lean body mass (logBLBM) and baseline log-transformed body surface area (logBBSA) correlated with baseline log-transformed body weight (logBWGT); baseline log-transformed aspartate aminotransferase (logBAST) correlated with baseline log-transformed alanine aminotransferase (logBALT); baseline log-transformed total protein (logBTOTP) correlated with baseline log-transformed albumin (logBALB) (Figure S4). Thus, among

the listed covariates only logBWGT, logBALT and logBALB were selected for further covariate search procedure.

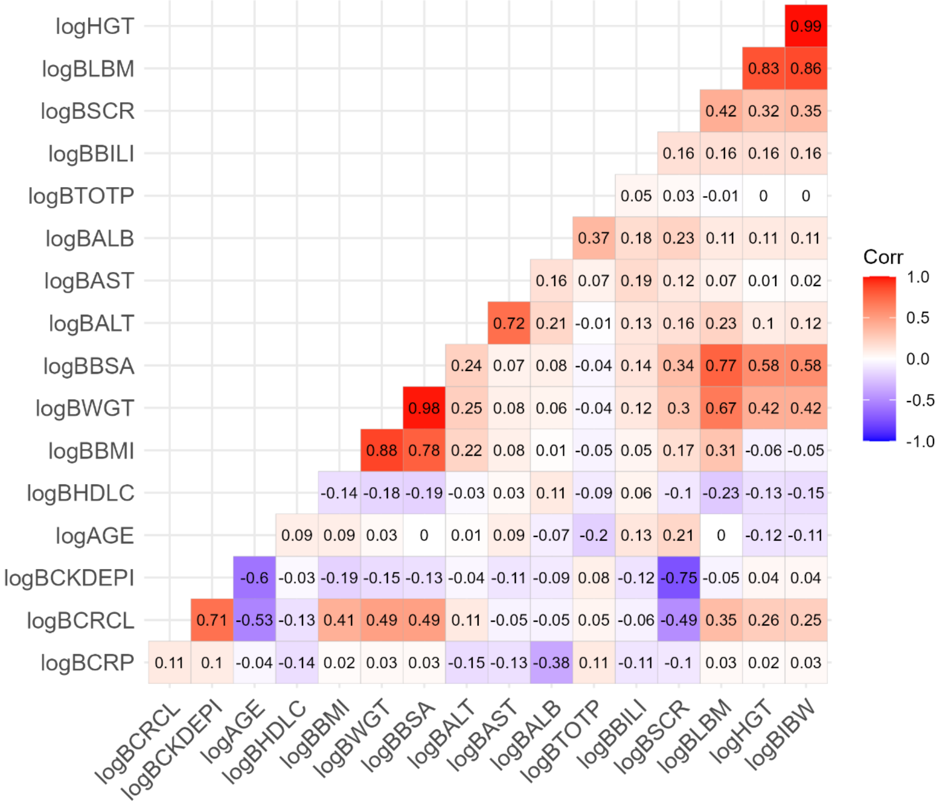


*Figure S4. Pearson correlation plot between logarithmically transformed covariates of CREDO 1 and CREDO 2 studies.*

## Long-term outcomes for olokizumab, adalimumab and placebo

The primary objective of CREDO 4 study was to evaluate the long-term safety and tolerability of OKZ 64 mg q2w or q4w in subjects with moderately to severely active rheumatoid arthritis (RA) who previously completed 24 weeks of double-blind treatment in core phase III studies (CREDO 1, 2 or 3). The long-term efficacy, immunogenicity, the physical function and quality of life of subjects received long-term treatment with OKZ were assessed in this study as well.

CREDO 4 study has long-term outcomes published [9]. However standalone it carried a set of limitations since it was an uncontrolled open-label extension of the core studies. Thus, virtual clinical trial simulations based on an adequate PK/PD model were performed to compare the long-term efficacy of OKZ 64 mg q2w and q4w dosing with that of ADLM and placebo, each with concomitant MTX.

To overview the long-term treatment outcome variability for ADLM and PBO a systematic literature review was conducted in PubMed to gather all sources reporting ADLM efficacy in MTX-IR subjects (populations of patients matching the baseline demographic and

clinical characteristics of the CREDO patient population). The review adhered to the Preferred Reporting Items for Systematic Reviews and Meta-Analyses (PRISMA) guidelines, ensuring a thorough and transparent approach to data collection [27].

Studies were selected according to the following inclusion criteria:

- Phase III randomized controlled trials (RCTs)
- Interventions involving reference ADLM only (biosimilars excluded)
- Patients with moderate to severe RA and MTX-IR or active RA condition
- Outcomes reported for DAS28-CRP and/or CDAI at week 24 (mandatory) and week 48 or 52 (if available)
- Non-responder imputation (NRI), LOCF or as observed methodology accepted
- Populations primarily from Europe or globally representative cohorts

Studies were excluded if they:

- Focused on biosimilars or switching from reference ADLM to biosimilars without detailed outcomes for ADLM control arm
- Included non-MTX-IR populations, early RA, or monotherapy arms only
- Lacked extractable efficacy data on relevant endpoints

To acquire the PubMed database for ADLM efficacy outcomes a following search string was applied:

*(("adalimumab"[Mesh] OR adalimumab[tiab])*
*AND ("rheumatoid arthritis"[Mesh] OR "rheumatoid arthritis"[tiab])*
*AND (randomized[tiab] OR "randomized controlled trial"[Publication Type] OR "clinical trial"[PublicationType]) AND ("inadequate response"[tiab] OR "MTX-IR"[tiab] OR "DMARD inadequate response"[tiab] OR "incomplete response"[tiab] OR "inadequate efficacy"[tiab] OR "biosimilar"[tiab] OR "CT-P17"[tiab] OR "switch"[tiab] OR "switching"[tiab] OR "reference adalimumab" OR "active RA"[tiab]))*

The search string was run in August 2025 and provided 95 items given additional filters applied for the publications in English, for studies in humans and dated 2005-2025. The application of selection criteria ensured the following steps of data selection (Table S3):

*Table S3. PRISMA flow diagram for ADLM published outcomes identification*

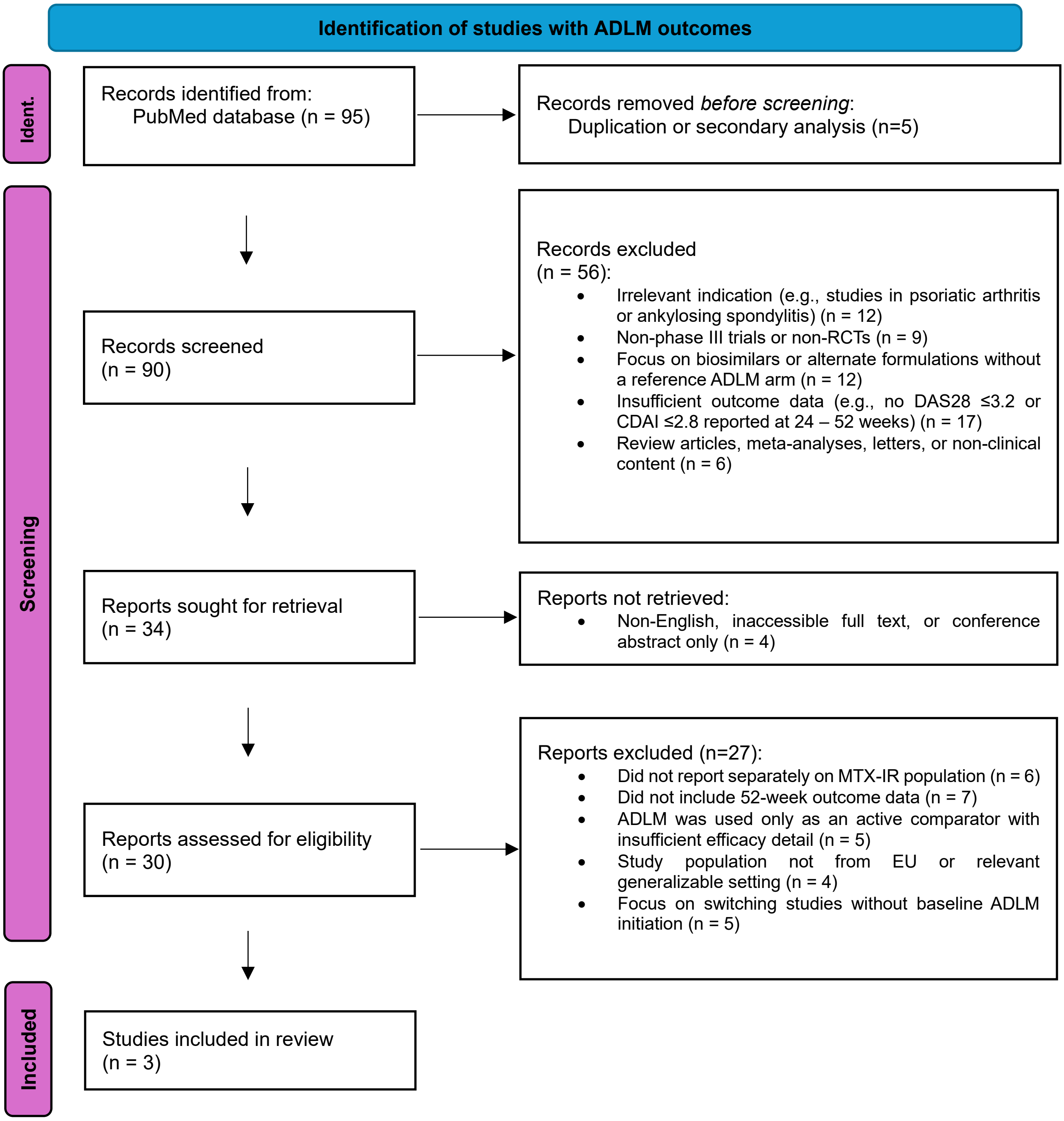


All finally selected ADLM studies:

- Included ADLM reference (Humira®) either alone or as active comparator.
- Included MTX-IR patients as part of the study design.
- Reported clinical efficacy outcomes at 52 weeks, especially DAS28-CRP ≤ 3.2 and/or DAS28-CRP ≤ 2.6 and/or CDAI ≤ 2.8.

Thus, the selected list of ADLM publications included the following references [21,22,24]:

- Taylor PC, Keystone EC, van der Heijde D, Weinblatt ME, Del Carmen Morales L, Reyes Gonzaga J, et al. Baricitinib versus placebo or adalimumab in rheumatoid arthritis. *N Engl J Med*. 2017;376(7):652–62. doi:10.1056/NEJMoa1608345
- Furst DE, Jaworski J, Wojciechowski R, Wiland P, Dudek A, Krogulec M, et al. Efficacy and safety of switching from reference adalimumab to CT-P17 (100 mg/ml): 52-week randomized, double-blind study in rheumatoid arthritis. *Rheumatology (Oxford)*. 2022;61(4):1385–95. doi:10.1093/rheumatology/keab460
- Combe B, Kivitz A, Tanaka Y, van der Heijde D, Simon JA, Baraf HSB, et al. Filgotinib versus placebo or adalimumab in patients with rheumatoid arthritis and inadequate response to methotrexate: a phase III randomized clinical trial. *Ann Rheum Dis*. 2021;80(7):848–58. doi:10.1136/annrheumdis-2020-219214

For an exhaustive overview of the ADLM outcomes and their dynamics, additional references, all of them among the 95 initially screened records, were included. One additional reference was selected for shorter-term ADLM data (up to 6 months, [35]) but related to the same clinical trial that was analyzed in [22].

- Kay J, Jaworski J, Wojciechowski R, Wiland P, Dudek A, Krogulec M, et al. Efficacy and safety of biosimilar CT-P17 versus reference adalimumab in subjects with rheumatoid arthritis: 24-week results from a randomized study. *Arthritis Res Ther*. 2021;23(1):51. doi:10.1186/s13075-020-02394-7

Another additional reference was identified for the long-term ADLM data (up to 12 months, [23]) from DE019 clinical trial:

- Keystone EC, Breedveld FC, van der Heijde D, van Vollenhoven RF, Emery P, Smolen JS, et al. Achieving comprehensive disease control in patients with early and established rheumatoid arthritis treated with adalimumab plus methotrexate versus methotrexate alone. *RMD Open*. 2017;3(2):e000445. doi:10.1136/rmdopen-2017-000445

Finally, an additional reference was identified for the long-term outcomes for PBO (MTX) at 52 weeks in MTX-IR patients [28]. As this study evaluated tocilizumab versus placebo and was therefore not retrieved by the adalimumab-specific search string, it was identified by manual searching outside the PubMed query and included solely as a source of long-term placebo (MTX) outcomes:

- Kremer JM, et al. Tocilizumab Inhibits Structural Joint Damage in Rheumatoid Arthritis Patients with Inadequate Responses to Methotrexate. Arthritis & Rheumatism 2011; 63: 609–621. doi: 10.1002/art.30158

Patients' baseline characteristics in the published ADLM+MTX and PBO studies were similar to those in CREDO 2 (Table S4). The published ADLM+MTX and PBO study outcomes were summarized in Table S5.

*Table S4. Summary table for published ADLM+MTX and PBO studies baseline characteristics vs. those in CREDO 2 ADLM+MTX arm*

| **Reference** | **N** | **Female, %** | **Age, mean±sd** | **Baseline CRP, mg/L mean±sd** | **Baseline DAS28-CRP, mean±sd** | **Duration of RA diagnosis, years mean±sd** |
|---|---|---|---|---|---|---|
| CREDO 2 (ADLM arm) | 460 | 80.3 | 54.3 ± 12.3 | 18.6 ± 18.5 | 5.88 ± 0.87 | 7.4 ± 7.5 |
| Combe 2021 | 325 | 81.8 | 53 ± 12.9 | - | 5.7 ± 0.9 | 8.0 ± 7.4 |
| Kay 2021; Furst 2022* | 324 | 82 | 50.5 ± 9.7 | 11.0 ± 19.1 | 5.54 ± 0.85 | 6.6 ± 6.8 |
| Taylor 2017 | 330 | 76 | 53 ± 12 | - | 5.8 ± 0.9 | 10.0 ± 9.0 |
| Keystone 2017 (DE019) | 207 | 76 | 56.1 ± 13.5 | 18.0 ± 23.0 | 5.6 ± 0.9 | 11.0 ± 9.2 |
| Kremer 2011 (PBO) | 393 | 83 | 51.3 ± 12.4 | 22 ± 25 | 6.5 ± 1.0 | 9.0 (0.5 - 44.3)** |

*The paper Furst 2022 describes continuation of the ADLM+MTX study was described in Kay 2021.

** In Kremer 2011

*Table S5. Summary table for ADLM+MTX and PBO published outcomes*

| **Reference** | **N patients** | **Endpoint** | **Mean score ±SD at Baseline** | **Week of treatment** | **Rate, %** | **Missing data handling method** |
|---|---|---|---|---|---|---|
| Combe 2021 | 325 | DAS28-CRP ≤ 3.2 | 5.7 ± 0.9 | 12 | 43 | NRI |
| Combe 2021 | 325 | DAS28-CRP ≤ 3.2 | 5.7 ± 0.9 | 24 | 51 | NRI |
| Combe 2021 | 325 | DAS28-CRP ≤ 3.2 | 5.7 ± 0.9 | 52 | 59 | NRI |
| Combe 2021 | 325 | CDAI≤2.8 | 39.2 ± 11.5 | 12 | 6 | NRI |
| Combe 2021 | 325 | CDAI≤2.8 | 39.2 ± 11.5 | 24 | 17 | NRI |
| Combe 2021 | 325 | CDAI≤2.8 | 39.2 ± 11.5 | 52 | 23 | NRI |
| Kay 2021 | 324 | DAS28-CRP ≤ 2.6 | 5.54 ± 0.85 | 12 | 33 | NRI |
| Kay 2021 | 324 | DAS28-CRP ≤ 2.6 | 5.54 ± 0.85 | 16 | 36.4 | NRI |
| Kay 2021 | 324 | DAS28-CRP ≤ 2.6 | 5.54 ± 0.85 | 20 | 45.1 | NRI |
| Kay 2021 | 324 | DAS28-CRP ≤ 2.6 | 5.54 ± 0.85 | 24 | 48.5 | NRI |

| | | | | | | |
|---|---|---|---|---|---|---|
| Furst 2022 | 153 | DAS28-CRP ≤ 2.6 | 5.54 ± 0.8 | 52 | 52.3 | NRI |
| Kay 2021 | 324 | CDAI≤2.8 | 38.7 ± 10.8 | 12 | 13 | NRI |
| Kay 2021 | 324 | CDAI≤2.8 | 38.7 ± 10.8 | 16 | 19.4 | NRI |
| Kay 2021 | 324 | CDAI≤2.8 | 38.7 ± 10.8 | 20 | 26.2 | NRI |
| Kay 2021 | 324 | CDAI≤2.8 | 38.7 ± 10.8 | 24 | 26.5 | NRI |
| Furst 2022 | 153 | CDAI≤2.8 | 38.3 ± 10.1 | 52 | 34 | NRI |
| Taylor 2017 | 330 | DAS28-CRP ≤ 3.2 | 5.8 ± 0.9 | 12 | 35 | LOCF |
| Taylor 2017 | 330 | DAS28-CRP ≤ 3.2 | 5.8 ± 0.9 | 24 | 48 | LOCF |
| Taylor 2017 | 330 | DAS28-CRP ≤ 3.2 | 5.8 ± 0.9 | 52 | 48 | LOCF |
| Keystone 2017 (DE019) | 207 | DAS28-CRP ≤ 3.2 | 5.6 ± 0.9 | 12 | 32.4 | LOCF |
| Keystone 2017 (DE019) | 207 | DAS28-CRP ≤ 3.2 | 5.6 ± 0.9 | 24 | 39.5 | LOCF |
| Keystone 2017 (DE019) | 207 | DAS28-CRP ≤ 3.2 | 5.6 ± 0.9 | 52 | 48.1 | LOCF |
| Keystone 2017 (DE019) | 207 | DAS28-CRP ≤ 2.6 | 5.6 ± 0.9 | 12 | 13.6 | LOCF |
| Keystone 2017 (DE019) | 207 | DAS28-CRP ≤ 2.6 | 5.6 ± 0.9 | 24 | 23.0 | LOCF |
| Keystone 2017 (DE019) | 207 | DAS28-CRP ≤ 2.6 | 5.6 ± 0.9 | 52 | 27.5 | LOCF |
| Kremer 2011 (PBO) | 393 | DAS28-CRP ≤ 3.2 | 6.5 ± 1.0 | 52 | 7.1 | NRI |

The outcomes of this analysis suggested that DAS28-CRP (Figure S5) as well CDAI endpoints (Figure S6) demonstrated substantial variability given the similar clinical study design for MTX-IR subjects.

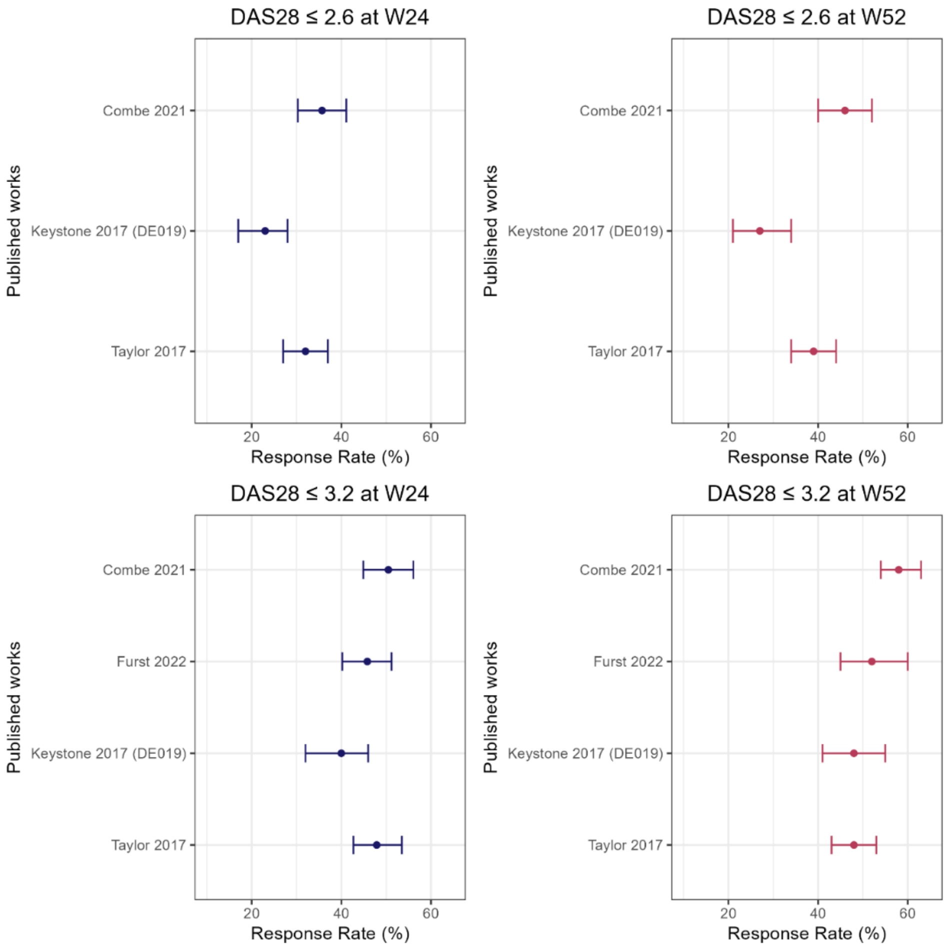


*Figure S5. ADLM and placebo treatment efficacy in published data: (upper panel) patients' proportion with remission (DAS28-CRP ≤ 2.6), (lower panel) patients' proportion with low disease activity (DAS28-CRP ≤ 3.2)*

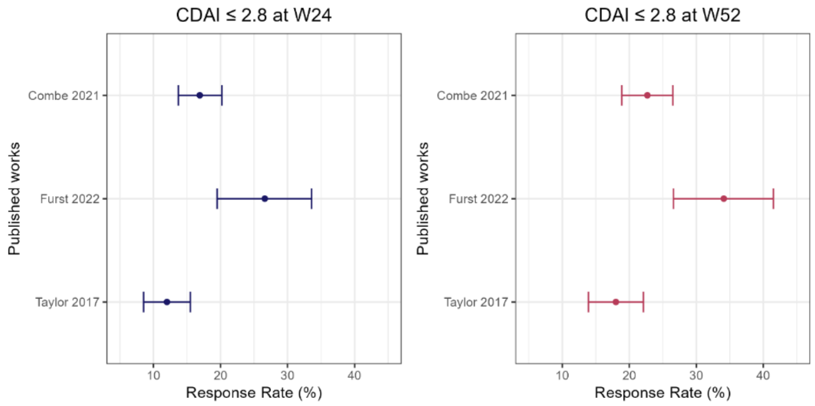


*Figure S6. ADLM treatment efficacy in published data: (left) patients' proportion with CDAI≤2.8 at week 24, (right) patients' proportion with CDAI≤2.8 at week 52*

## OKZ PK model development

For OKZ two compartment PK model, linear clearance and 1st order absorption after SC injection was selected. The random effects for V1 and CL and correlation term between the random effects were considered. Combined residual error model was applied.

The final PK model contained a correlation term between individual parameters of CL and V1 [41]. The Monolix ver. 2024R1 does not fully allow the user to fix correlation parameters for simulations. To overcome this issue, the final OKZ PK model was re-parameterized, and the correlation between random effects was modeled via scaled shared and independent components of between-subject variability of CL parameter:

$$V1_i = \exp\left(\log\left(V1_{pop}\right) + \eta_i^{V1}\right) \quad \text{(s1)}$$

$$CL_i = \exp\left(log\left(CL_{pop}\right) + scale * \eta_i^{V1} + \eta_i^{CL}\right) \quad \text{(s2)}$$

The reparametrized PK model was similar to the final PK model in terms of parameter estimates and AIC (Table S6). Thus, this updated PK model was taken for all tested PK/PD models.

*Table S6. Parameters of the alternatively parameterized final OKZ PK model.*

| Parameter | Value | SE | RSE (%) | Description |
|---|---|---|---|---|
| **Fixed Effects** | | | | |
| **$k_a$** ($d^{-1}$) | 0.22 | 0.011 | 4.85 | Absorption rate from SC depo compartment |
| **log($V_1$)** | 1.44 | 0.036 | 2.46 | Log of central compartment volume typical value (in L) |
| **log(CL)** | -1.73 | 0.011 | 0.653 | Log of central clearance typical value (in L/d) |
| **Q** (L/d) | 0.58 | 0.045 | 7.78 | Between compartment clearance |
| **$V_2$** (L) | 2.32 | 0.092 | 3.98 | Peripheral compartment volume |
| **F** | 0.88 | 0 (Fixed) | 0 (Fixed) | Bioavailability coefficient for SC injection |
| **$\beta_{V1\_logBALT}$** | -0.22 | 0.057 | 25.3 | |
| **$\beta_{V1\_logBWGT}$** | 1.16 | 0.12 | 10.2 | |

| | | | | |
|---|---|---|---|---|
| $\beta_{Cl\_logBALB}$ | -1.02 | 0.17 | 16.8 | |
| $\beta_{Cl\_logBHDLC}$ | -0.34 | 0.05 | 14.8 | |
| $\beta_{Cl\_logBWGT}$ | 0.49 | 0.062 | 12.6 | |
| **scale** | 0.13 | 0.022 | 16.4 | Special coefficient to consider correlation between random effects for V1 and CL |
| **Random Effects** | | | | |
| $\omega_{V1}$ | 0.72 | 0.027 | 3.78 | SD in normal distribution (with mean=0) of $\eta_i^{V1}$ |
| $\omega_{Cl}$ | 0.42 | 0.011 | 2.57 | SD in normal distribution (with mean=0) of $\eta_i^{CL}$ |
| **Residual Error** | | | | |
| **a** (μg/ml) | 0.091 | 0.01 | 11.1 | Coefficient of constant part in residual error model |
| **b** | 0.23 | 0.0033 | 1.43 | Coefficient of proportional part in residual error model |
| **AIC** | 35176.5 | | | |

A set of baseline covariate effects were considered in the final PK model (log-transformed serum albumin, high-density lipoprotein cholesterol and body weight (BWGT) for CL; log-transformed alanine aminotransferase and BWGT for V1).

As PPP&D sequential approach was executed for PK/PD modeling [30], population PK parameters must be fixed for PK/PD model building.

## DAS28-CRP PK/PD model development

### DAS28-CRP Base model development

Based on graphical exploration of DAS28-CRP data (see PD data exploration), $I_{max}$-type model was suggested to describe relationship between OKZ concentration and DAS28-CRP changes.

$$\frac{dR}{dt} = k_{out} \cdot R0 \cdot \left(1 - \frac{Imax \cdot (DMARD + Conc_{OKZ})}{IC50 + (DMARD + Conc_{OKZ})}\right) - k_{out} \cdot R \quad \text{(s3)}$$

In the case of ADLM with concomitant MTX treatment the model was modified as follows:

$$\frac{dR}{dt} = k_{out} \cdot R0 \cdot \left(1 - \frac{Imax \cdot PADLM}{IC50 + PADLM}\right) - k_{out} \cdot R \qquad (s4)$$

In equation (s4) the same *IC50* parameter value (derived from OKZ treatment data description) was used. Thus, the parameter *PADLM* in equation (s4) was in units of OKZ concentration (the same for parameter *DMARD*).

The selection of optimal model was based on AIC, parameters identifiability (RSE < 50%) and shrinkage for PD parameters (η-shrinkage < 51%). When the optimal base model was selected random effects were tested for existing of correlation between them.

It was found that simple $I_{max}$ model could not describe slower component of DAS28-CRP change observed in data. Thus, hypothetical dependence of $I_{max}$ parameter on time since treatment started was suggested in the model.

$$I_{max,eff}(t) = \frac{I_{max}}{1 + w_1 \cdot \exp(-\beta_1 \cdot t)} \qquad (s5)$$

In the model $I_{max,eff}$ gradually growths from $I_{max}/(1+w_1)$ to $I_{max}$ value with time. The rate of this growth depends on parameter $\beta_1$ (1/day).

To check the significance of possible difference in the parameters $w_1$ and $\beta_1$ between OKZ and ADLM treatments the categorical covariate ADLMTRTc (1 – for ADLM treatment; 0 – otherwise) effects were tested in the models.

Overall, 9 models with different structural and statistical components were tested. Extensive search for the optimal structural model, random effects and residual error ensured base model selection. Thus, the selected base PK/PD model for DAS28-CRP was $I_{max}$ model with time dependent $I_{max}$ value, random effects for $R_0$ and $I_{max}$ and correlation term between the random effects. The model was fully identifiable with RSE for all parameters <50% (Table S7) and $\omega^2$-based η-shrinkage for $R_0$ and $I_{max}$ < 51%. No significant effect of ADLM treatment on $I_{max}$ time dependent parameters was found.

*Table S7. Parameters of the DAS28-CRP base PK/PD model.*

| Parameter | Value | SE | RSE (%) |
|---|---|---|---|
| **Fixed Effects** | | | |
| **$R_0$ [DAS28-$CRP_{Baseline}$]** | 5.88 | 0.0276 | 0.469 |
| **$k_{out}$** ($d^{-1}$) | 0.133 | 0.0073 | 5.49 |
| **$I_{max}$** | 0.523 | 0.0105 | 2.01 |
| **$IC_{50}$** (μg/ml) | 0.507 | 0.0192 | 3.78 |
| **DMARD** (μg/ml) | 0.629 | 0.0241 | 3.84 |
| **PADLM** (μg/ml) | 3.76 | 0.500 | 13.30 |
| **$w_1$** | 0.924 | 0.0127 | 1.38 |
| **$\beta_1$** | 0.022 | 0.00125 | 5.68 |
| **Random Effects** | | | |
| **$\omega_{R0}$** | 0.125 | 0.00409 | 3.29 |
| **$\omega_{Imax}$** | 0.871 | 0.0333 | 3.82 |
| **Correlations** | | | |
| **Corr (R0, Imax)** | -0.257 | 0.0466 | 18.1 |
| **Residual Error** | | | |
| **a** | 0.668 | 0.00622 | 0.931 |

## DAS28-CRP final model development

The following list of covariates was taken for covariate search: logAGE, sex, region (RGN), alternative region separation – Latin America/Others (RGN2), ethnicity, logBWGT, logBCKDEPI, logBALB, logBALT, logBHDLC, logBCRP and logBBILI. Stepwise covariate search was applied to develop optimal covariate models. Overall, 13 covariate models were tested under covariate search for DAS28-CRP PK/PD model. The final PK/PD model includes the following covariate dependences: logBCRP for *R0* parameter and RGN2 (region= Latin America) categorical covariate for $I_{max}$ parameter (Table S8).

*Table S8. Parameters of the DAS28-CRP final PK/PD model*

| Parameter | Value | SE | RSE (%) |
|---|---|---|---|
| **Fixed Effects** | | | |
| **R0 [DAS28-$CRP_{Baseline}$]** | 5.89 | 0.0262 | 0.445 |
| **$\beta_{R0\_logBCRP}$** | 0.0649 | 0.00366 | 5.64 |
| **$k_{out}$** ($d^{-1}$) | 0.141 | 0.00996 | 7.08 |

| | | | |
|---|---|---|---|
| **$I_{max}$** | 0.495 | 0.0111 | 2.23 |
| **$\beta_{Imax\_RGN2=1}$** | 0.381 | 0.0644 | 16.9 |
| **$IC_{50}$** (μg/ml) | 0.576 | 0.0263 | 4.57 |
| **DMARD** (μg/ml) | 0.72 | 0.0537 | 7.46 |
| **PADLM** (μg/ml) | 4.18 | 0.715 | 17.1 |
| **$w_1$** | 0.916 | 0.0184 | 2.01 |
| **$\beta_1$** | 0.0224 | 0.00128 | 5.69 |
| **Random Effects** | | | |
| **$\omega_{R0}$** | 0.1 | 0.00384 | 3.83 |
| **$\omega_{Imax}$** | 0.858 | 0.0325 | 3.79 |
| **Correlations** | | | |
| **Corr (R0, Imax)** | -0.421 | 0.0489 | 11.6 |
| **Residual Error** | | | |
| **a** | 0.67 | 0.00614 | 0.917 |

According to diagnostic plots below, the model provided adequate description of the data (Figures S7 – S11).

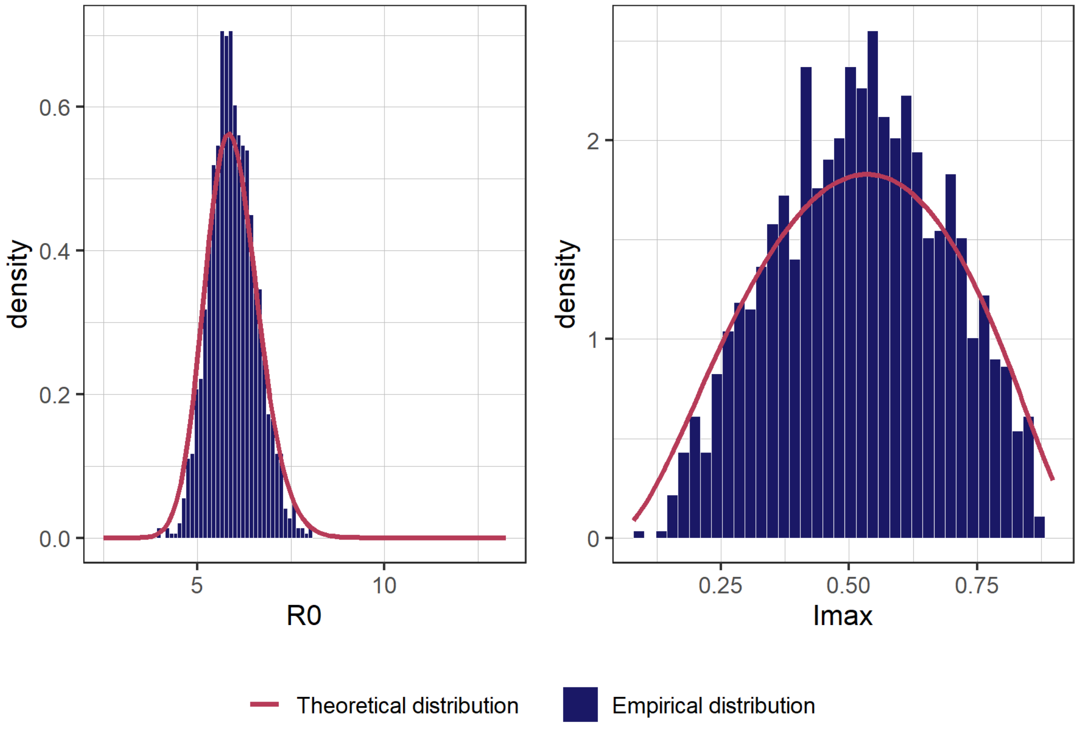


*Figure S7. Individual parameter values distribution in the DAS28-CRP final PK/PD model. Dark blue bars – histograms of empirical Bayes estimates (EBE), red line – theoretical distribution. η-shrinkage was 18.9% and 17% for $R_0$ and $I_{max}$, respectively.*

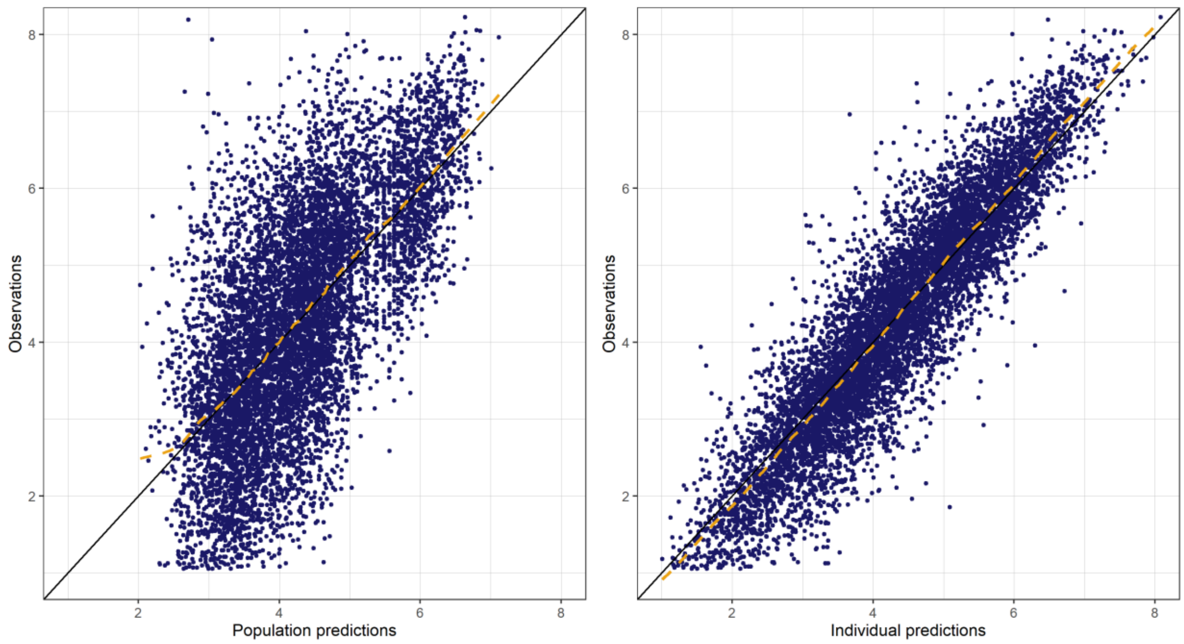


*Figure S8. Goodness-of-fit plots of the DAS28-CRP final PK/PD model: observation vs. individual predictions. Black lines on plots are diagonal lines (y = x), dark blue dots are individual points, yellow dashed line is a spline approximation of the points*

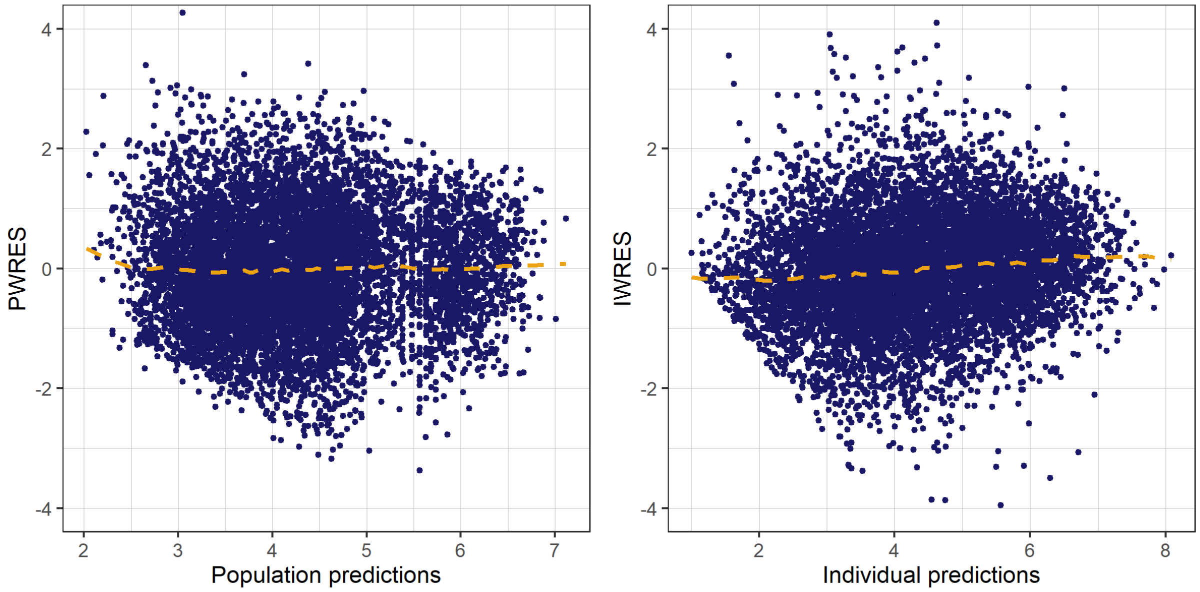


*Figure S9. Goodness-of-fit plots of the DAS28-CRP final PK/PD model: population (left) and individual (right) weighted residuals vs. predictions plots. Black lines on plots are diagonal lines (y = 0), dark blue dots are individual points, yellow dashed line is a spline approximation of the points.*

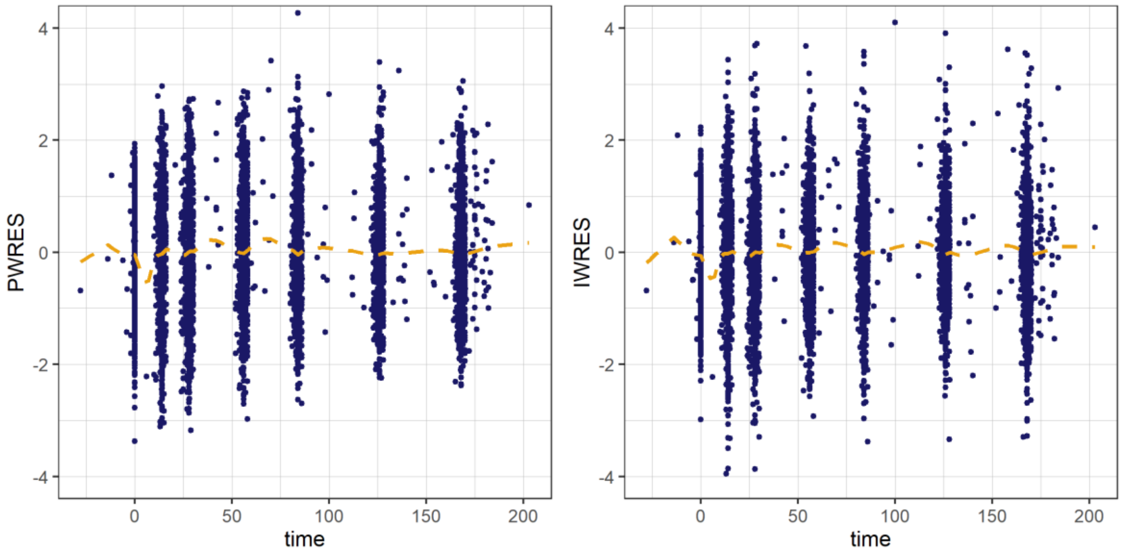


*Figure S10. Goodness-of-fit plots of the DAS28-CRP final PK/PD model: population (left) and individual (right) weighted residuals vs. time (days). Black lines on plots are diagonal lines (y = 0), dark blue dots are individual points, yellow dashed line is a spline approximation of the points.*

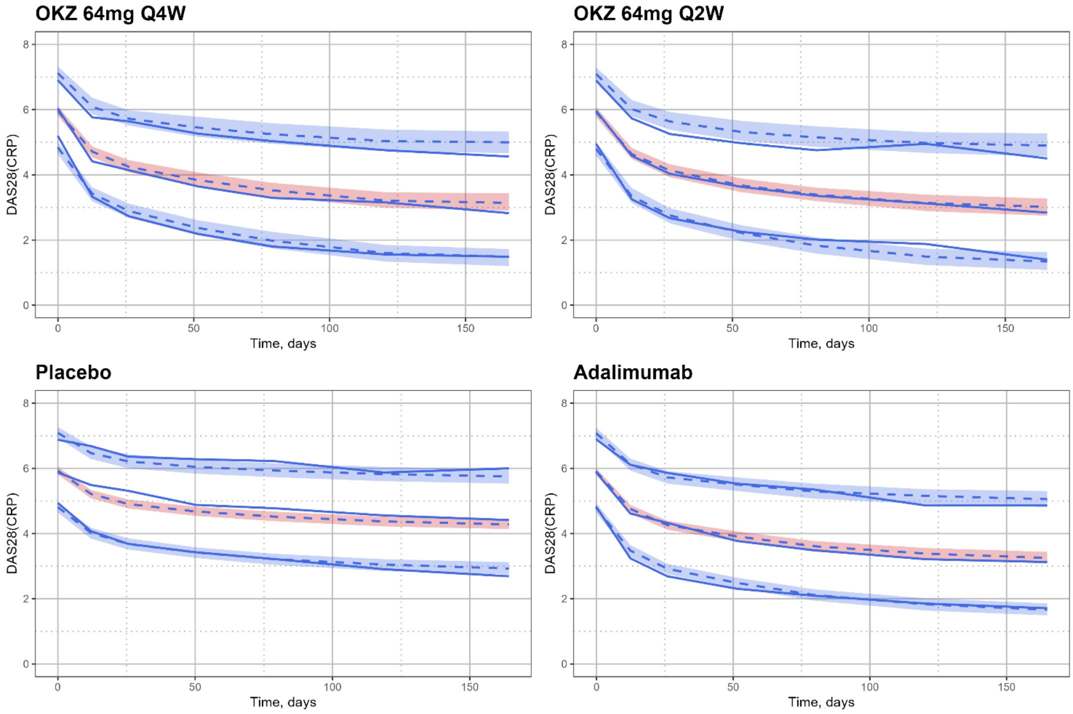


*Figure S11. Goodness-of-fit plots of the DAS28-CRP final PK/PD model: visual predictive check plots. Solid lines – 5th, 50th and 95th percentiles of experimental data; dashed lines and corresponding ribbons are theoretical predictions with 95% CI.*

# CDAI PK/PD model development

## CDAI Base model development

Based on graphical exploration of CDAI data (see PD data exploration) and the previously developed DAS28-CRP PK/PD model, Imax-type model was suggested to describe relationship between OKZ concentration and CDAI changes. Dependent variable (CDAI score) was square-root transformed for more efficient parameter optimization procedure while all further graphical and table diagnostic incorporated back-transformed values (equations s6 - s8).

$$\frac{d}{dt}(Rsqrt) = k_{out} \cdot Rsqrt0 \cdot \left(1 - \frac{Imax \cdot (DMARD + Conc_{OKZ})}{IC50 + (DMARD + Conc_{OKZ})}\right) - k_{out} \cdot Rsqrt \qquad \text{(s6)}$$

In the case of ADLM with concomitant MTX treatment the model was modified as follows:

$$\frac{d}{dt}(Rsqrt) = k_{out} \cdot Rsqrt0 \cdot \left(1 - \frac{Imax \cdot PADLM}{IC50 + PADLM}\right) - k_{out} \cdot Rsqrt \qquad \text{(s7)}$$

$$R = Rsqrt^2 \qquad \text{(s8)}$$

The model includes random effects for $Rsqrt_0$ and $I_{max}$ parameters and correlation between the random effects was tested. Hypothetical dependence of $I_{max}$ parameter on time since treatment started was suggested in the CDAI model as well:

$$I_{max,eff}(t) = \frac{I_{max}}{1 + w_1 \cdot \exp(-\beta_1 \cdot t)} \qquad \text{(s9)}$$

$I_{max,eff}$ gradually growths from $I_{max}/(1+w_1)$ to $I_{max}$ value with time. The rate of this growth depends on parameter $\beta_1$ (1/day).

To acquire proper parameter identifiability PADLM parameter was fixed to fPADLM*$IC_{50}$ value where fPADLM was tested to be 5, 10 or 20. To check the significance of possible difference in the parameters $I_{max}$, $w_1$ and $\beta_1$ between OKZ and ADLM treatments the categorical covariate ADLMTRTc (1 - for ADLM treatment; 0 - otherwise) effects were tested in the models.

The selection of optimal model was based on AIC, parameters identifiability (RSE < 50%) and shrinkage for PD parameters (η-shrinkage < 51%). When the optimal base model was selected random effects were tested for existing of correlation between them.

Overall, 33 models with different structural and statistical components were tested. Extensive search for the optimal structural model, random effects and residual error led to the base PK/PD model for CDAI which includes time dependent $I_{max}$ value, random

effects for $Rsqrt_0$ and $I_{max}$ and correlation term between the random effects and combined type of residual error. The statistically significant difference in $I_{max}$ parameter value for ADLM and OKZ/PBO treatments was considered. The parameter fPADLM=10 was shown to be optimal. The model was fully identifiable with RSE for all parameters <50% and $\omega^2$-based η-shrinkage for $Rsqrt_0$ and $I_{max}$ < 51% (Table S9). Diagnostic plots showed adequate description of the data (not shown here for the base model).

*Table S9. Parameters of the CDAI base PK/PD model.*

| Parameter | Value | SE | RSE (%) |
|---|---|---|---|
| **Fixed Effects** | | | |
| **$R_0$ [$CDAI_{Baseline}$]** | 6.21 | 0.0314 | 0.506 |
| **$k_{out}$ ($d^{-1}$)** | 0.0935 | 0.00442 | 4.73 |
| **$I_{max}$** | 0.454 | 0.0134 | 2.96 |
| **$IC_{50}$** (μg/ml) | 0.866 | 0.0257 | 2.97 |
| **DMARD** (μg/ml) | 1.84 | 0.163 | 8.88 |
| **fPADLM** | 10 | | |
| **$\beta_{Imax\_ADLMTRTc\_1}$** | 0.439 | 0.0766 | 17.4 |
| **$w_1$** | 1.21 | 0.0241 | 1.99 |
| **$\beta_1$** | 0.0255 | 0.0011 | 4.32 |
| **Random Effects** | | | |
| **$\omega_{R0}$** | 0.137 | 0.00419 | 3.06 |
| **$\omega_{Imax}$** | 1.05 | 0.0352 | 3.37 |
| **Correlations** | | | |
| **Corr ($R_0$, $I_{max}$)** | -0.271 | 0.0407 | 15 |
| **Residual Error** | | | |
| **a** | 2.07 | 0.121 | 5.88 |
| **b** | 0.214 | 0.00682 | 3.18 |

## CDAI Final model development

A set of covariate models were tested under covariate search for CDAI PK/PD model. In addition to the initially identified dependence of $I_{max}$ from ADLM treatment in the base model the resulting optimal model incorporated more covariates: logBCRP for $Rsqrt_0$ parameter, RGN2 (1 if region is Latin America; 0 - otherwise) categorical covariate for $I_{max}$ parameter and ADLM treatment (ADLMTRTc) on $I_{max}$ parameter. Thus, the optimal model structure was close to the one collected during DAS28-CRP PK/PD modeling (Table S10).

*Table S10. Parameters of the CDAI final PK/PD model.*

| Parameter | Value | SE | RSE (%) |
|---|---|---|---|
| **Fixed Effects** | | | |
| **$R_0$ [$CDAI_{Baseline}$]** | 6.21 | 0.0318 | 0.512 |
| **$\beta_{R0\_logBCRP}$** | 0.0204 | 0.00437 | 21.4 |
| **$k_{out}$** ($d^{-1}$) | 0.0911 | 0.00589 | 6.47 |
| **$I_{max}$** | 0.438 | 0.0146 | 3.32 |
| **$\beta_{Imax\_ADLMTRTc\_1}$** | 0.418 | 0.0818 | 19.6 |
| **$\beta_{Imax\_RGN2=1}$** | 0.347 | 0.0758 | 21.9 |
| **$IC_{50}$** (µg/ml) | 0.992 | 0.00258 | 0.26 |
| **DMARD** (µg/ml) | 2.07 | 0.285 | 13.8 |
| **fPADLM** | 10 | | |
| **$w_1$** | 1.18 | 0.0451 | 3.82 |
| **$\beta_1$** | 0.0244 | 0.00147 | 6.05 |
| **Random Effects** | | | |
| **$\omega_{R0}$** | 0.134 | 0.00442 | 3.29 |
| **$\omega_{Imax}$** | 1.05 | 0.0379 | 3.61 |
| **Correlations** | | | |
| **Corr ($R_0$, $I_{max}$)** | -0.289 | 0.0448 | 15.5 |
| **Residual Error** | | | |
| **a** | 2.14 | 0.124 | 5.78 |
| **b** | 0.21 | 0.00683 | 3.25 |

This model improved fit to data by 35.65 according to AIC (vs. CDAI Base PK/PD model) and had all parameters identified. According to diagnostic plots, the model provided adequate description of the data (Figures S12 – S16).

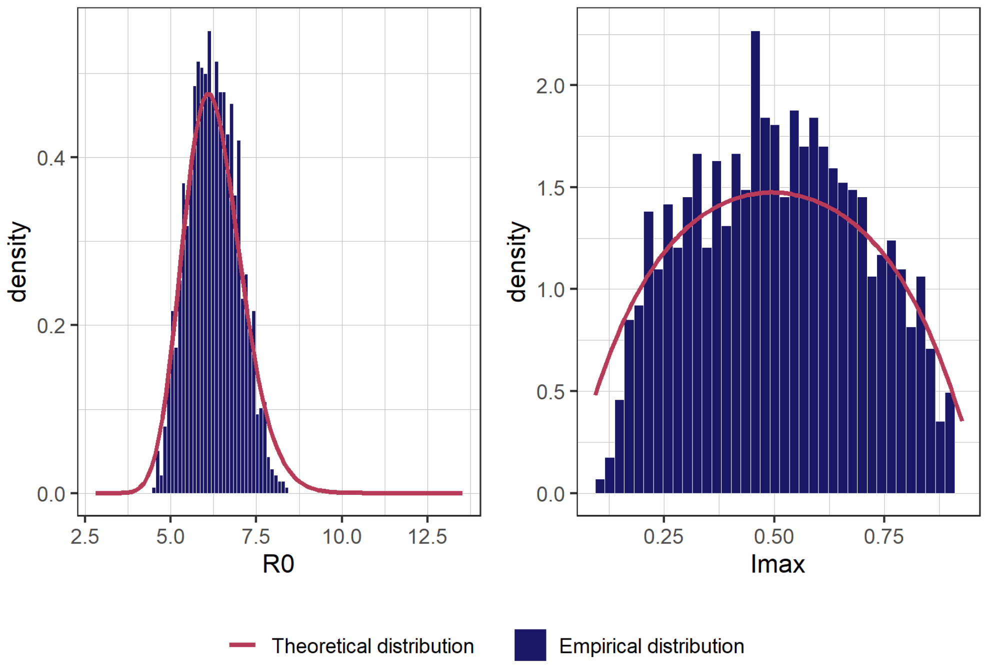


*Figure S12. Individual parameter distributions in the CDAI final PK/PD model. Dark blue bars – histograms of EBE, red line – theoretical distribution: η-sh($R_0$) = 15.3%, η-sh($I_{max}$) = 19.1%.*

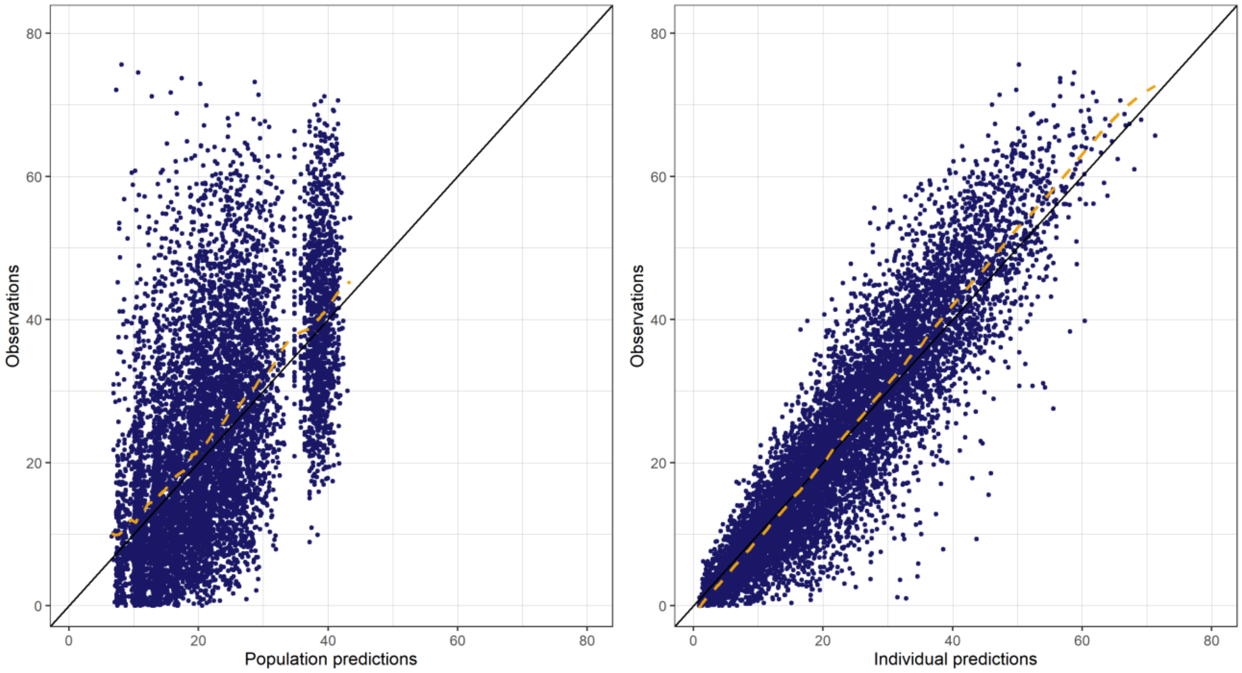


*Figure S13. Goodness-of-fit plots of the CDAI final PK/PD model: observation vs. individual predictions. Black lines on plots are diagonal lines (y = x), dark blue dots are individual points, yellow dashed line is a spline approximation of the points.*

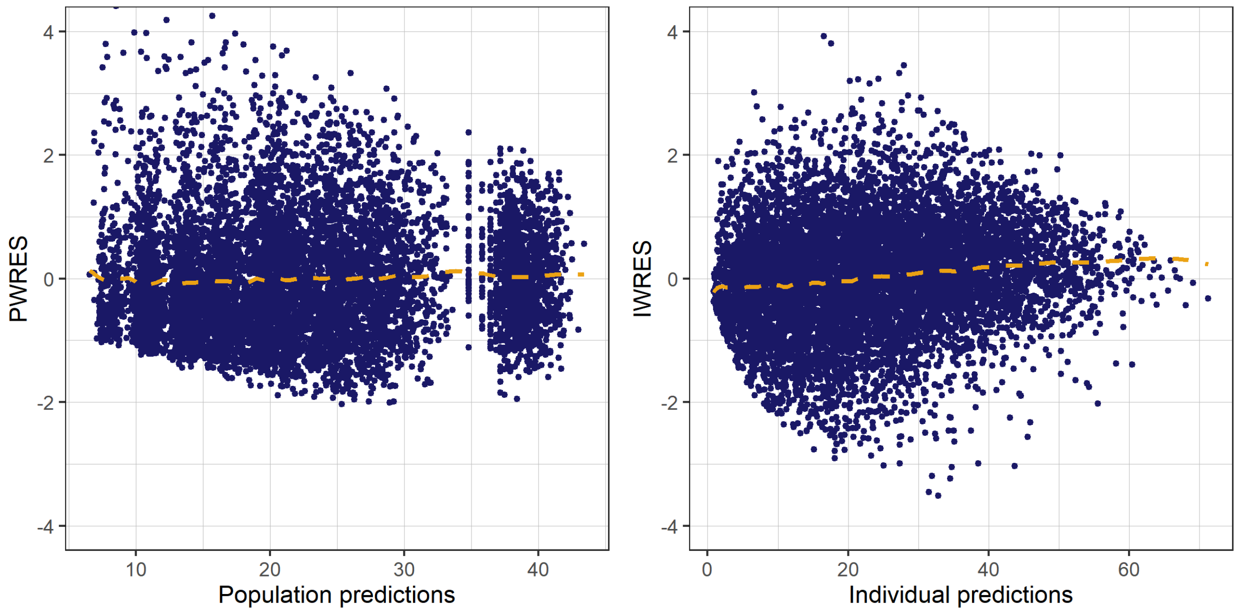


*Figure S14. Goodness-of-fit plots of the CDAI final PK/PD model: population (left) and individual (right) weighted residuals vs. predictions plots. Black lines on plots are diagonal lines (y = 0), dark blue dots are individual points, yellow dashed line is a spline approximation of the points.*

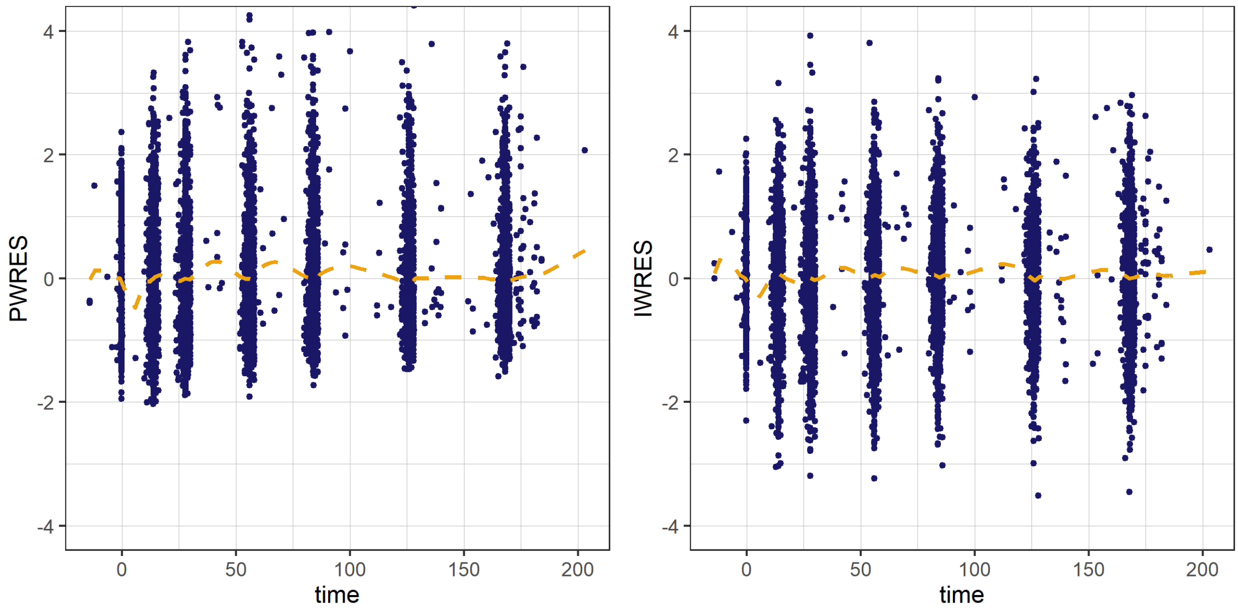


*Figure S15. Goodness-of-fit plots of the CDAI final PK/PD model: population (left) and individual (right) weighted residuals vs. time (days). Black lines on plots are diagonal lines (y = 0), dark blue dots are individual points, yellow dashed line is a spline approximation of the points.*

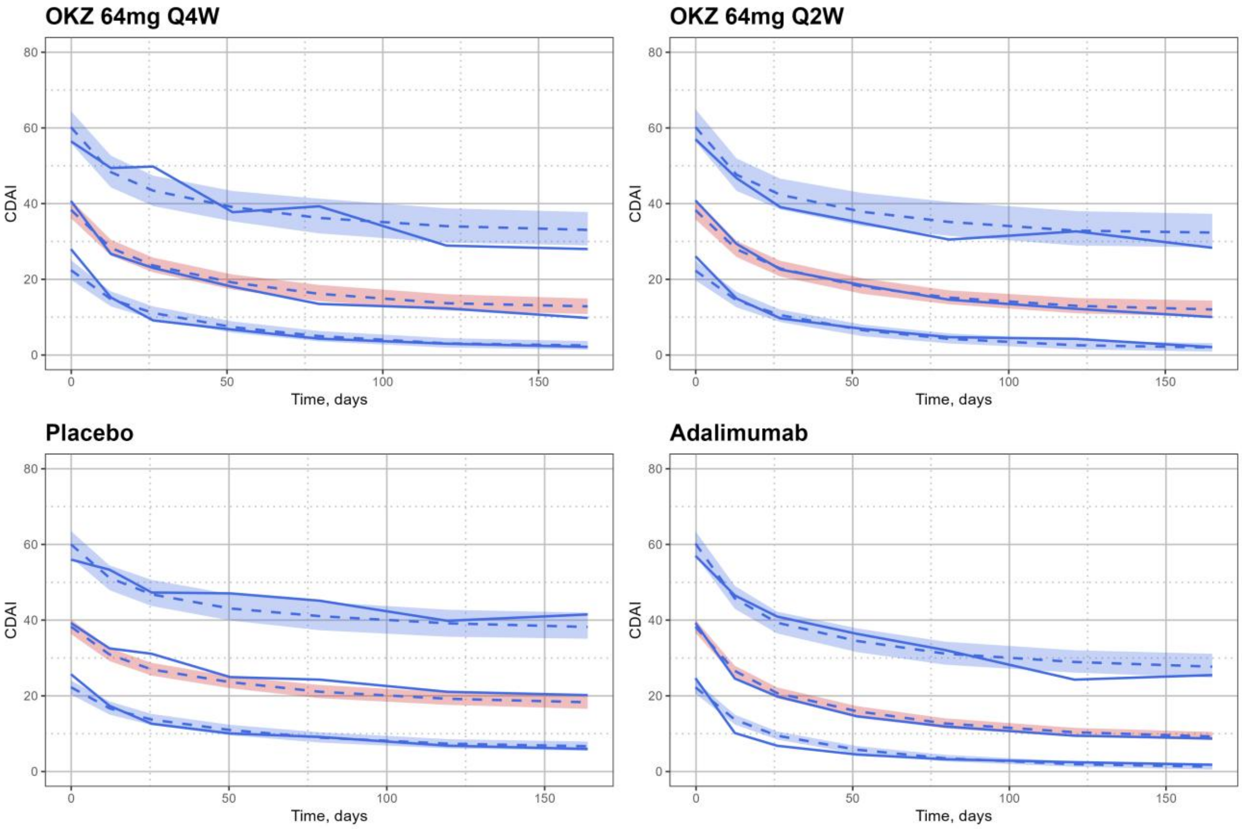


*Figure S16. Goodness-of-fit plots of the CDAI final PK/PD model: visual predictive check plots. Solid lines – 5th, 50th and 95th percentiles of experimental data; dashed lines and corresponding ribbons are theoretical predictions with 95% CI.*

## PK/PD model predictions' sensitivity to covariate change

Forest plots were generated to assess the impact of statistically significant covariates for PD parameters on clinical indexes (Figure S17 and S18). Particularly: 200 replicas of population parameters were simulated, accounting for variance-covariance matrix of parameter estimates from Monolix project of the final PK/PD models. For each replica of the population parameters, each significant covariate was fixed at 5th, 50th, 95th percentiles of respective distribution, while other significant covariates were randomly sampled from PK/PD modeling dataset. 1000 subjects were simulated per each fixed covariate value and treatment group, and random effects were generated using parameters for IIV.

Using the final DAS28-CRP/CDAI PK/PD models at baseline, week 12, 24 and 52 outcomes were simulated for PBO, SC OKZ 64 mg q4w and OKZ 64 mg q2w, and ADLM 40 mg q2w for each of the 1000 simulated subjects. DAS28-CRP/CDAI values were simulated from the final DAS28-CRP (CDAI) PK/PD model with residual error. For each population of the 1000 sampled subjects, percentage of patients with DAS28≤3.2 (CDAI≤2.8) were calculated. Clinical endpoints for SC OKZ 64 mg q4w and OKZ 64 mg

q2w dosage, ADLM 40 mg q2w and PBO regimens for the 5th and 95th percentiles of a given covariate were compared to those in patients with the median covariate value (or baseline level in case of categorical covariates). Results over all simulated replicas of population parameters and for the 5th and 95th percentiles of the given covariate were summarized with median 90% CI.

During the covariate effect assessment via the forest plots the highest effects were identified for logBCRP and the region (Latin America or not) in the DAS28-CRP final PK/PD model (Figure S17). Despite the statistical significance of all identified covariates, their impact on PD effect size was modest, suggesting the lack of pharmacological covariate relevance. The effects of the PK model covariates on PD outcomes were negligible.

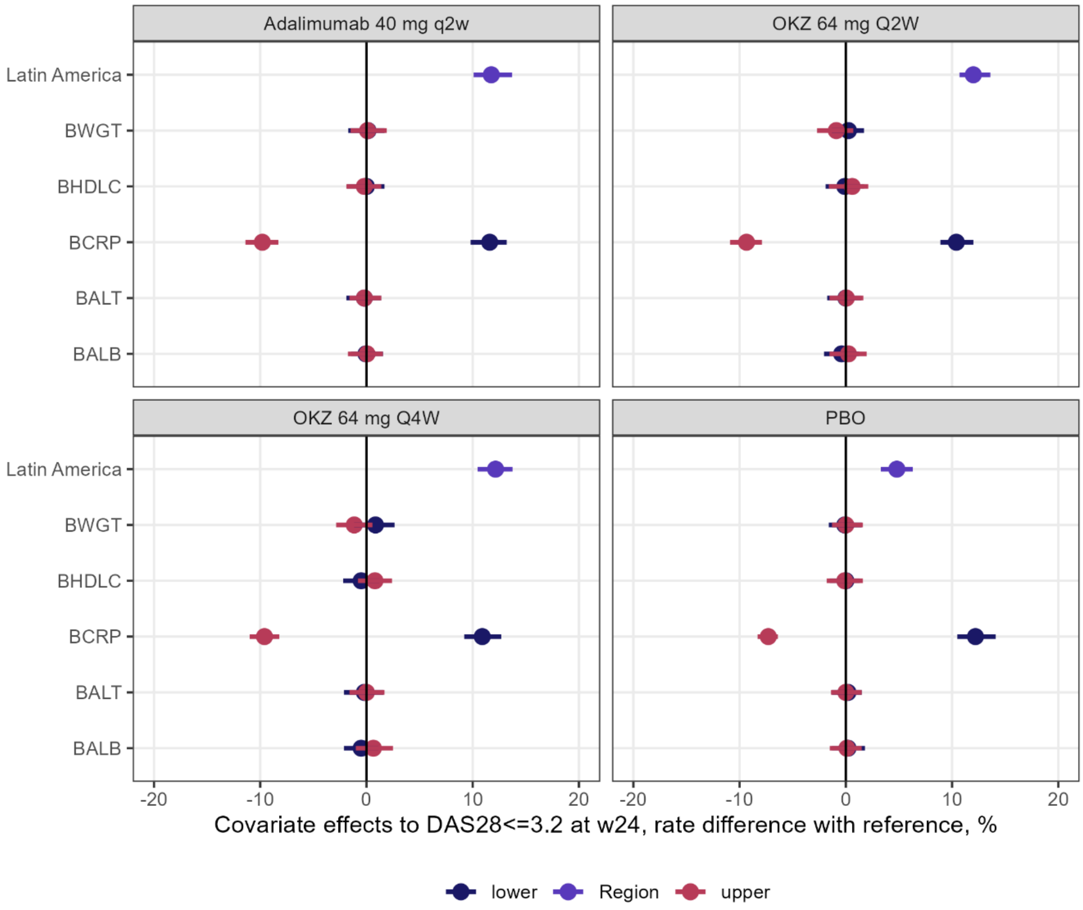


*Figure S17. Covariate impact on DAS28-CRP outcome at 24 weeks of treatment for all considered treatments.*

During the covariate effect assessment, minor effects were identified for logBCRP and the region (Latin America or not) in the CDAI final PK/PD model (Figure S18). Despite

the statistical significance of all identified covariates, their impact on PD effect size was not profound suggesting the lack of pharmacological relevance.

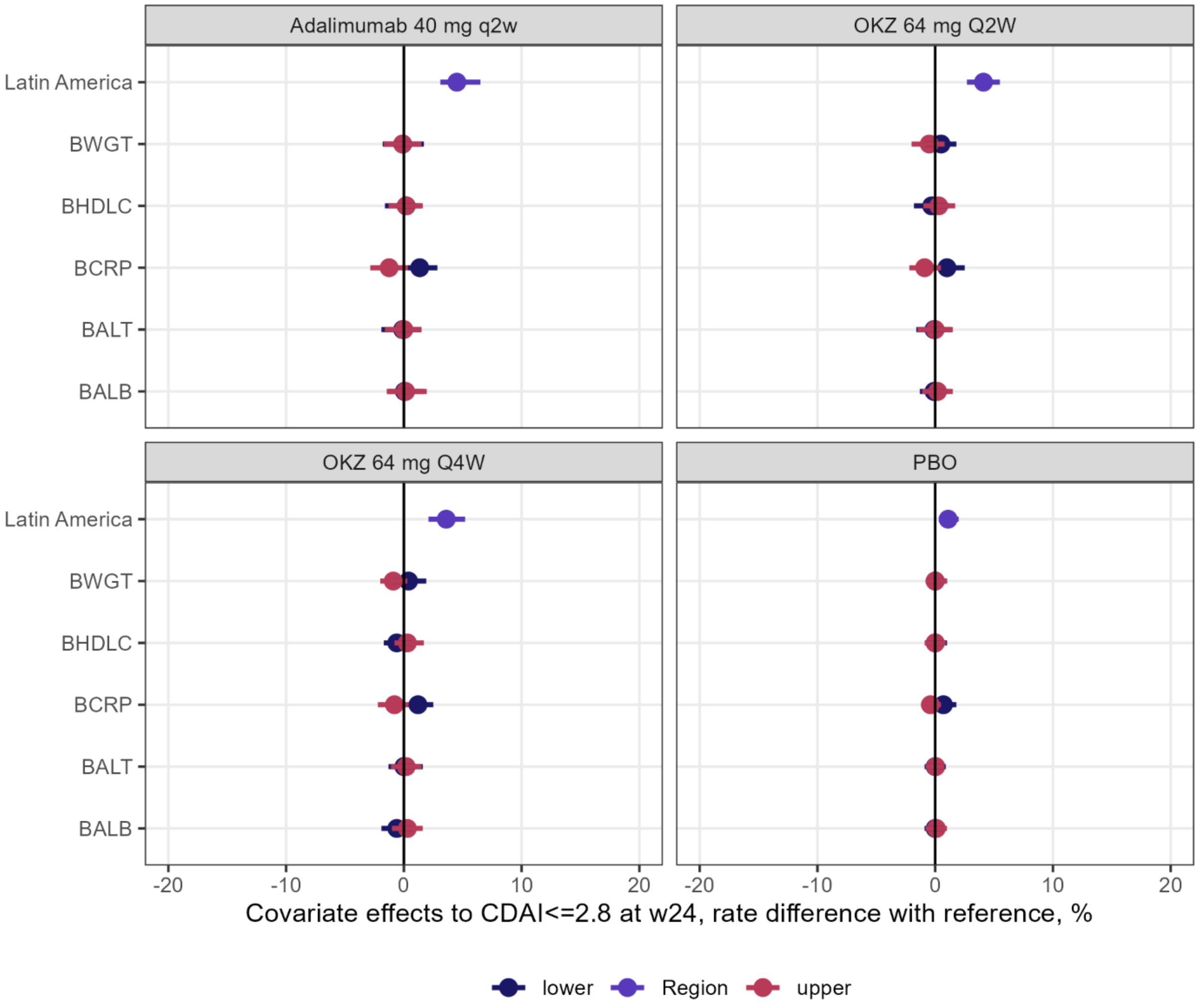


*Figure S18. Covariate impact on CDAI outcome at 24 weeks of treatment for all considered treatments.*

Additionally, PBO-correction was applied to the data and respective forest plots were presented (Figure S19 – S20). PBO-correction makes all the covariate impact on the outcomes of OKZ and ADLM treatments negligible.

Finally, current sensitivity analysis suggests that given statistical significance of selected covariates for PK/PD models their pharmacological relevance was not achieved due to the minor covariate effect on the simulated outcomes.

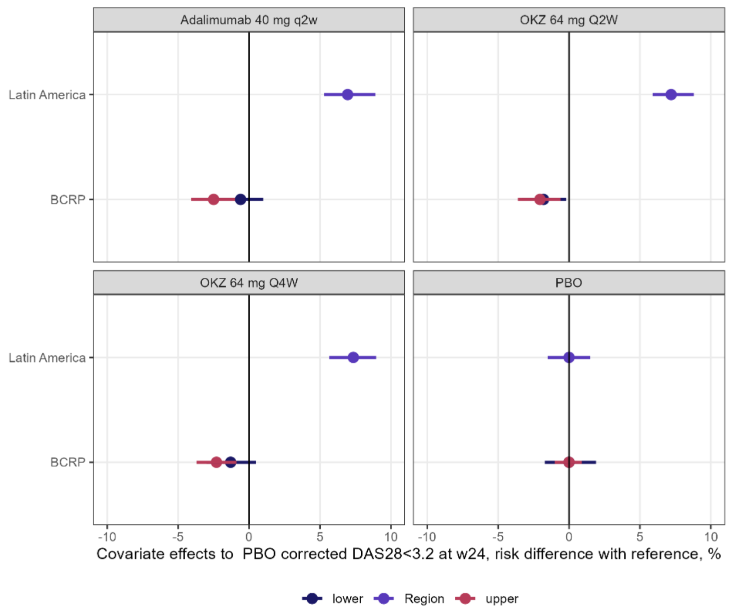


*Figure S19. Covariate impact on DAS28-CRP outcome at 24 weeks of treatment for all considered treatments. PBO-corrected outcomes.*

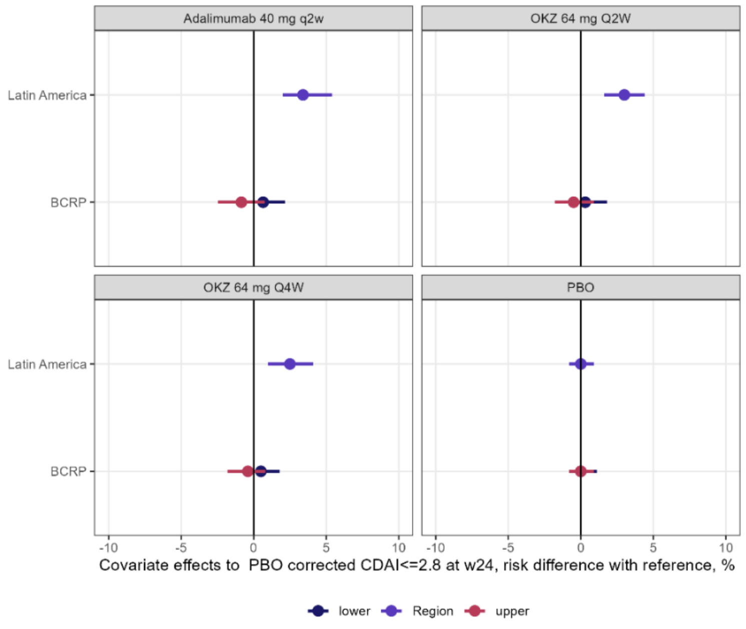


*Figure S20. Covariate impact on CDAI outcome at 24 weeks of treatment for all considered treatments. PBO-corrected outcomes.*

## The subjects' exclusion from the full dataset

The full source dataset includes n = 427 and n = 1645 subjects from CREDO1 and CREDO2 study, respectively. The final PKPD modeling dataset includes n = 258 and n = 1056 subjects from CREDO1 and CREDO2 study, respectively. The subjects were excluded from OKZ treatment arms mainly due to the absence of PK measurements or due to the absence of any post-treatment PD measurements (Table S11).

*Table S11. Subjects' numbers by study and arm in the Source dataset and the final PKPD modeling dataset*

| Study | Treatment | Subject number in the Source dataset | Subject number in the Final PKPD dataset |
|---|---|---|---|
| CREDO 1 | OKZ q4w | 142 | 63 |
| CREDO 1 | OKZ q2w | 143 | 56 |
| CREDO 1 | PBO | 142 | 139 |
| CREDO 2 | OKZ q4w | 477 | 186 |
| CREDO 2 | OKZ q2w | 463 | 171 |
| CREDO 2 | PBO | 243 | 239 |
| CREDO 2 | ADLM | 462 | 460 |

Comparison of baseline characteristics between included and non-included OKZ-treated patients was shown in *Table S12*. The median age and time from RA diagnosis, female proportion, baseline CRP, DAS28 and CDAI values etc. are very similar between included and non-included OKZ patients. Thus, the comparison outcomes suggest the absence of selection bias.

The final modeling dataset remains representative which is confirmed by very similar dynamics of DAS28(CRP) response rate were calculated from the Source and the PKPD modeling dataset, respectively (Figure S21).

*Table S12. Demographic and other baseline characteristics of OKZ treatment arms of CREDO 1 CREDO 2 studies: Included into the PKPD dataset vs. Non-Included.*

| **Characteristic** | **Statistic** | **Included N=476** | **Non-Included N=749** |
|---|---|---|---|
| Age (years) | | | |
| | n | 476 | 749 |
| | Mean (SD) | 51.42 (11.898) | 53.70 (12.090) |
| | Median (Q1, Q3) | 52.00 (44.00, 59.00) | 55.00 (46.00, 63.00) |

| Characteristic | Statistic | Included<br>N=476 | Non-Included<br>N=749 |
|---|---|---|---|
| | Min, Max | 19.0, 83.0 | 20.0, 82.0 |
| Sex | | | |
| | Female | 382 ( 80.3%) | 579 ( 77.3%) |
| | Male | 94 ( 19.7%) | 170 ( 22.7%) |
| Race | | | |
| | Asian | 12 ( 2.5%) | 4 ( 0.5%) |
| | Black or African American | 25 ( 5.3%) | 10 ( 1.3%) |
| | Other | 56 ( 11.8%) | 47 ( 6.3%) |
| | White | 383 ( 80.5%) | 688 ( 91.9%) |
| Ethnicity | | | |
| | Hispanic or Latino | 171 ( 35.9%) | 186 ( 24.8%) |
| | Not Hispanic or Latino | 305 ( 64.1%) | 563 ( 75.2%) |
| Region of Participation | | | |
| | Latin America | 148 ( 31.1%) | 164 ( 21.9%) |
| | Rest of the World | 222 ( 46.6%) | 382 ( 51.0%) |
| | Western | 106 ( 22.3%) | 203 ( 27.1%) |
| | | | |
| Time from RA diagnosis (years) | | | |
| | n | 476 | 749 |
| | Mean (SD) | 7.05 (6.981) | 7.93 (7.531) |
| | Median (Q1, Q3) | 4.73 (2.00, 9.72) | 5.55 (1.96, 11.52) |
| | Min, Max | 0.3, 38.9 | 0.3, 40.5 |
| Intolerance to >=15 mg/week MTX Dose | | | |
| | No | 426 ( 89.5%) | 651 ( 86.9%) |
| | Yes | 50 ( 10.5%) | 98 ( 13.1%) |
| BMI (kg/m^2) | | | |
| | n | 476 | 749 |
| | Mean (SD) | 28.17 (6.158) | 28.15 (6.011) |
| | Median (Q1, Q3) | 27.15 (24.00, 31.34) | 27.34 (23.92, 31.25) |
| | Min, Max | 17.0, 62.2 | 14.9, 58.0 |
| History of Extra-Articular Features | | | |
| | No | 445 ( 93.5%) | 699 ( 93.3%) |
| | Yes | 31 ( 6.5%) | 50 ( 6.7%) |
| C-Reactive Protein (mg/L) | | | |
| | n | 476 | 749 |
| | Mean (SD) | 21.25 (22.858) | 18.78 (19.066) |
| | Median (Q1, Q3) | 13.00 (7.00, 27.25) | 12.00 (7.00, 24.00) |
| | Min, Max | 1.0, 175.0 | 1.0, 151.0 |

| Characteristic | Statistic | Included<br>N=476 | Non-Included<br>N=749 |
|---|---|---|---|
| Tender Joint 68 Count | | | |
| | n | 475 | 749 |
| | Mean (SD) | 24.54 (12.674) | 23.10 (12.010) |
| | Median (Q1, Q3) | 22.00 (15.00, 30.95) | 20.00 (14.21, 29.00) |
| | Min, Max | 6.0, 68.0 | 6.0, 68.0 |
| Swollen Joint 68 Count | | | |
| | n | 475 | 749 |
| | Mean (SD) | 15.54 (8.312) | 14.57 (7.348) |
| | Median (Q1, Q3) | 13.00 (9.50, 20.00) | 13.00 (9.00, 17.00) |
| | Min, Max | 4.1, 60.0 | 5.2, 58.0 |
| DAS28 (CRP) | | | |
| | n | 465 | 740 |
| | Mean (SD) | 5.97 (0.839) | 5.86 (0.802) |
| | Median (Q1, Q3) | 6.01 (5.36, 6.55) | 5.84 (5.33, 6.38) |
| | Min, Max | 3.3, 8.0 | 3.0, 8.3 |
| DAS28 (ESR) | | | |
| | n | 460 | 732 |
| | Mean (SD) | 6.65 (0.881) | 6.51 (0.878) |
| | Median (Q1, Q3) | 6.64 (6.09, 7.29) | 6.53 (5.94, 7.13) |
| | Min, Max | 3.8, 8.8 | 3.8, 9.0 |
| Clinical Disease Activity Index | | | |
| | n | 453 | 718 |
| | Mean (SD) | 41.00 (11.296) | 39.07 (10.864) |
| | Median (Q1, Q3) | 40.10 (32.90, 48.50) | 38.25 (31.00, 45.77) |
| | Min, Max | 9.9, 69.3 | 9.0, 68.3 |
| Simplified Disease Activity Index | | | |
| | n | 453 | 718 |
| | Mean (SD) | 43.13 (11.761) | 40.95 (11.256) |
| | Median (Q1, Q3) | 42.40 (34.50, 51.60) | 39.70 (32.82, 48.16) |
| | Min, Max | 11.0, 74.9 | 10.8, 75.2 |
| HAQ-DI Total Score | | | |
| | n | 465 | 740 |
| | Mean (SD) | 1.71 (0.598) | 1.70 (0.550) |
| | Median (Q1, Q3) | 1.75 (1.38, 2.12) | 1.75 (1.38, 2.00) |
| | Min, Max | 0.0, 3.0 | 0.0, 3.0 |
| Patient's Assessment of Disease Activity (VAS) (mm) | | | |
| | n | 465 | 740 |

| Characteristic | Statistic | Included N=476 | Non-Included N=749 |
|---|---|---|---|
| | Mean (SD) | 67.25 (19.499) | 67.94 (19.408) |
| | Median (Q1, Q3) | 69.00 (57.00, 81.00) | 70.00 (56.00, 82.00) |
| | Min, Max | 1.0, 100.0 | 2.0, 100.0 |
| Pain (VAS) (mm) | | | |
| | n | 465 | 740 |
| | Mean (SD) | 67.53 (20.255) | 68.28 (19.929) |
| | Median (Q1, Q3) | 71.00 (57.00, 83.00) | 71.00 (56.00, 83.00) |
| | Min, Max | 0.0, 100.0 | 0.0, 100.0 |
| Phys. Assmnt. of Disease Activity (VAS) (mm) | | | |
| | n | 456 | 719 |
| | Mean (SD) | 67.41 (15.054) | 66.67 (15.989) |
| | Median (Q1, Q3) | 68.00 (59.00, 77.00) | 68.00 (57.00, 78.00) |
| | Min, Max | 17.0, 100.0 | 9.0, 100.0 |

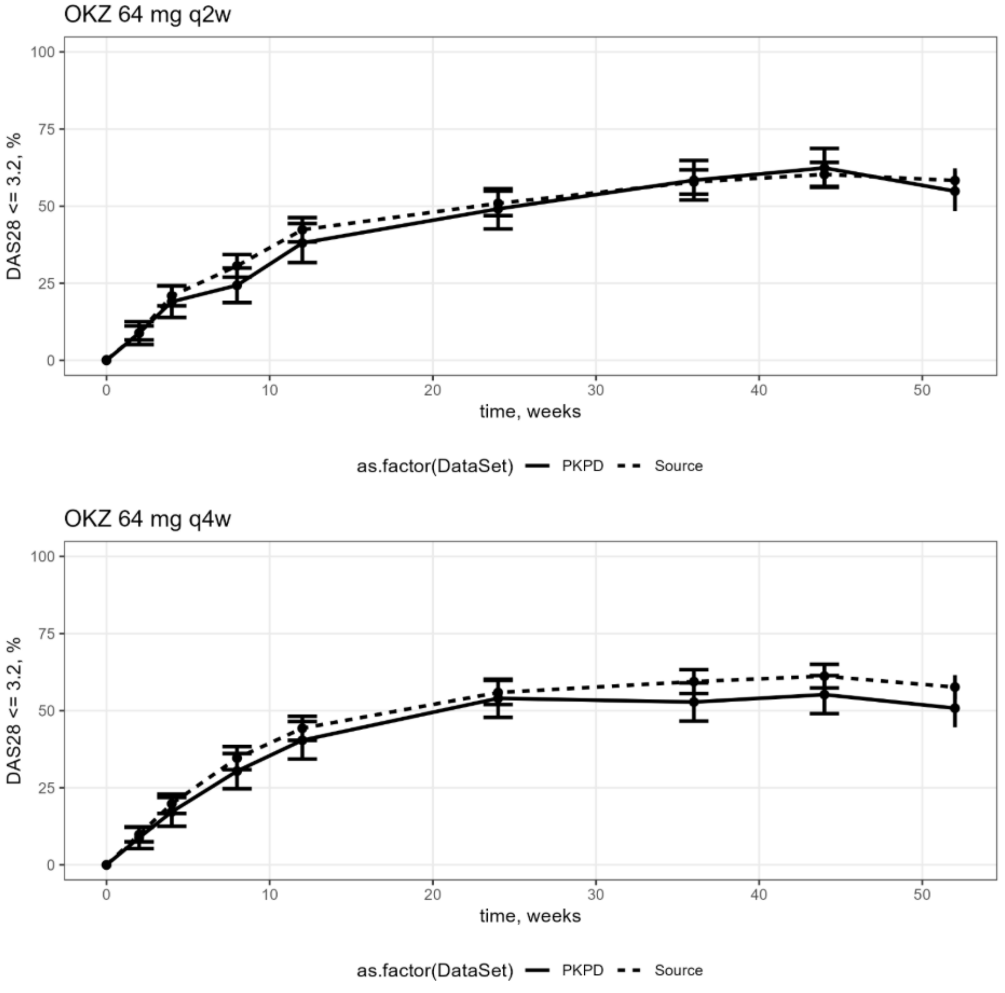


*Figure S21. DAS28(CRP) response rate dynamics for OKZ treatment arms were calculated from source dataset (dashed lines) and the final PKPD modeling dataset (solid lines).*
